# Rhenium isotopes reveal enhanced rock organic carbon oxidation over the Toarcian Oceanic Anoxic Event

**DOI:** 10.1038/s41467-026-71533-6

**Published:** 2026-04-16

**Authors:** Madeleine Stow, Alexander J. Dickson, Julie Prytulak, Mathieu Dellinger, Victoria Alcock, Stephen P. Hesselbo, Geoff M. Nowell, Robert G. Hilton

**Affiliations:** 1 Department of Earth Sciences, University of Oxford, South Parks Road, Oxford, UK; 2 Centre of Climate, Ocean and Atmosphere, Department of Earth Sciences, Royal Holloway University of London, Egham, Surrey UK; 3 Department of Earth Sciences, University of Durham, South Road, Durham, UK; 4 Department of Earth, Ocean and Atmospheric Sciences, University of British Columbia, Vancouver, Canada; 5 Environnements Dynamiques et Territoires de la Montagne (EDYTEM), CNRS – Université Savoie Mont-Blanc, Le Bourget du Lac, France; 6 Camborne School of Mines, Department of Earth and Environmental Sciences, University of Exeter, Penryn, Cornwall UK; 7 School of Geography and the Environment, University of Oxford, South Parks Road, Oxford, UK

**Keywords:** Carbon cycle, Palaeoclimate

## Abstract

Weathering plays a central role in the geological carbon cycle. Silicate mineral weathering is invoked as a stabilizing feedback on CO_2_ emissions, for example from volcanism during the emplacement of Large Igneous Provinces. However, modern-day studies show weathering can emit CO_2_ during oxidation of rock organic carbon (OC_petro_) in sedimentary rocks and function as a positive feedback on climate warming. Here we measure the rhenium isotope composition (δ^187^Re) of Early Jurassic marine sediments to explore how OC_petro_ oxidation rates changed during warming across the Toarcian Ocean Anoxic Event (T-OAE). We find a 0.22 ± 0.10‰ decrease in δ^187^Re values during the T-OAE, with mass balance modeling showing this can be explained by increased OC_petro_ weathering intensity on land associated with 6–7 °C of global warming. We estimate this could have delivered 7600–20,490 PgC to the oceans and atmosphere, demonstrating that chemical weathering does not simply act as a stabilizing feedback during hyperthermal events.

## Abstract (web summary)

Widespread volcanic eruptions caused rapid warming during the Early Jurassic. Rhenium isotopes reveal that this led to positive climate feedbacks as higher temperatures increased weathering of organic matter, releasing further greenhouse gases.

## Introduction

Earth’s climate is moderated by variations in the concentration of atmospheric CO_2_ set by the transfer of carbon between the solid Earth, oceans, biosphere and atmosphere. Over geological timescales, atmospheric sources of CO_2_ from volcanism and metamorphism^1,2^ are joined by the oxidative weathering of rock-derived organic carbon (OC_petro_) and sulfide minerals^3–6^. These CO_2_ inputs are removed from the atmosphere-ocean system by silicate mineral weathering coupled to carbonate precipitation^7^ and organic carbon burial^8^. The CO_2_ inputs are crucial to forcing changes in the surface carbon reservoirs^9^. In the case of volcanism, pulsed CO_2_ inputs have been linked to warming events over 10^5^–10^6^ year timescales^10^ with associated global environmental and ecological impacts^11^. Secular changes in volcanic CO_2_ emissions are proposed as an important control on the carbon cycle over 10 s to 100 s of millions of years^12^. In contrast, past rates of OC_petro_ oxidation and the associated CO_2_ emissions are uncertain^13,14^, even though modern global rates of OC_petro_ oxidation^15^ of 68 ^+16^/_−6_ MtC yr^−1^ are similar to those from volcanism^2^. Indeed, based on studies of modern weathering systems, we might expect OC_petro_ oxidation fluxes to change over geological time due to changes in global climate, tectonic regime, crustal lithology, and erosion rate, but OC_petro_ oxidation remains challenging to quantify.

Modern weathering studies suggest that OC_petro_ weathering and oxidation may act as a previously unrecognized climatic feedback, with increased CO_2_ release linked to enhanced erosion and warming^6,16^. This appears to counter the well-studied negative feedback offered by the weathering of silicate minerals by carbonic acid^7,17^. Empirical data and models suggest that physical weathering and erosion can supply large amounts of OC_petro_ to near-surface environments where chemical weathering and organic oxidation rates can be high^18^. Direct measurements of CO_2_ release from OC_petro_ oxidation have shown that rates increase > 2-fold for a 10 °C rise in local atmospheric temperatures^16,19^, thus acting as a potential positive feedback on warming. This process could play out at a global scale because there are large areas of the weathering zone, particularly in erosive settings, where OC_petro_ oxidation is incomplete^16,20^. Modern global OC_petro_ oxidation during weathering^15^ of 68 ^+16^/_−6_ MtC yr^−1^ occurs alongside the export of 43 ^+61^/_−25_ MtC yr^−1^ as incompletely weathered OC_petro_ contained in river sediments^20^. This implies an apparent OC_petro_ weathering intensity of 0.61 today^3,15^, meaning that changes in the rates of OC_petro_ oxidation linked to warming could increase global emission fluxes. Thus, it is critical to constrain how these feedbacks operated in the geological past to reach a quantitative understanding of Earth-system behavior under different equilibrium climate states, especially those that may be analogous to a warmer world in the near future. Whilst quantifying the solid residue of weathering using OC_petro_ across stratigraphic sections holds much promise^21,22^, we require other tracers of OC_petro_ oxidation to help provide a regional to global view of changes in oxidative weathering.

Rhenium isotopes (^185^Re and ^187^Re, with abundances of 37.4% and 62.6% respectively) could provide quantitative insight on oxidative weathering in the geological past^23–25^. While ^187^Re is a radiogenic isotope and decays to ^187^Os, the half-life of this decay is ~4 ×10^10^ years and therefore the isotopes of Re can be considered ‘stable’ on the timescales of weathering processes^23^. The association of Re with organic matter in sedimentary rocks^26,27^, its coupled loss during OC_petro_ oxidation^24,28^, and its solubility and mobility upon oxidation and transport in the dissolved loads of rivers^29–31^, provide rationale for using Re isotopes as a paleo-weathering proxy^25^. It has been demonstrated by several studies on modern weathering environments that Re isotopes are fractionated during oxidative weathering^23,32,33^. The δ^187^Re values (relative to the NIST 3143 standard, δ^187^Re = ((^187^Re/^185^Re)_sample_/(^187^Re/^185^Re)_NIST 3143_ − 1) × 1000) decrease as Re is lost during oxidative weathering, which is suggested to be caused by the preferential oxidative weathering of phases enriched in the heavy ^187^Re isotope^23,33^. Low weathering intensity results in the largest fractionation between river waters and weathering residues, while higher weathering intensity leads to river waters with lower δ^187^Re values that approach the starting rock composition^23,32,33^. Since rivers are the main source of Re to the oceans^25,29,34^, changes in oxidative weathering intensity and flux over geological time should change river δ^187^Re values and shift seawater Re isotopic compositions that may be recorded in marine sediments.

Here we assess oxidative weathering during the Early Jurassic Toarcian Oceanic Anoxic Event, T-OAE (also known as the Jenkyns Event, ~183 Ma). The T-OAE is characterized by a global perturbation in the carbon cycle, expressed as a positive carbon isotope excursion interrupted by a negative carbon isotope excursion (CIE) in marine and terrestrial sedimentary sections^35–39^. The duration^40^ of the CIE has been estimated between 0.3–1 Myr. The carbon cycle perturbation and negative CIE have variously been explained by the emplacement of the Karoo-Ferrar Large Igneous Province that released CO_2_ directly through degassing^41^ or by thermogenic alteration following magmatic intrusion into organic rich sediments^42,43^. Alternatively, increasing temperatures could have led to the destabilization and release of methane from gas hydrates^44^, enhanced fungal decomposition of organic matter^45^, or thawing permafrost^46^. Increased hydrological cycling^47,48^ is inferred to have driven increases in continental weathering rates, as indicated by radiogenic and stable isotope systems such as osmium and calcium^49–52^. Recent work on the T-OAE carbon budget highlighted an apparent missing source of carbon^10^, with the amount of CO_2_ released by the Karoo-Ferrar LIP during the T-OAE smaller than the amount required to drive associated changes in the partial pressure of atmospheric carbon dioxide (*p*CO_2_) and temperature in Earth System models. We hypothesize that CO_2_ release through oxidative weathering could have provided an additional source of CO_2_ during the T-OAE.

We present a Re isotope record from the Llanbedr (Mochras Farm) borehole, Cardigan Bay Basin, U.K., hereafter ‘Mochras’. Our data evidence a negative Re isotope excursion, which we evaluate using a mass balance model of the Re cycle. The only coherent explanation for the Re isotope excursion is that there was a notable increase in OC_petro_ oxidation rates during the transient warming event, confirming the presence of an unrecognized weathering feedback operating in the geological past.

## Results and discussion

### Sedimentary δ^187^Re records of the Toarcian OAE

The Mochras borehole captures continuous sedimentation from the Upper Triassic to Lower Jurassic in the Cardigan Bay Basin, an unrestricted, open marine setting^53^. The major lithologies are mudstone and limestone, which vary in proportion throughout the core^48^. The lack of major facies variations throughout the studied core interval supports the interpretation that there were no extreme variations in bottom water redox state or depositional environment that might have affected the local speciation of Re burial. We focus on the time period spanning the T-OAE, between core depths of ~850–775 m. The low abundance of Re in the Mochras core deposits makes isotopic measurements challenging, and we focus on 12 samples that span the T-OAE interval (Supplementary Data 1).

**Fig. 1:**
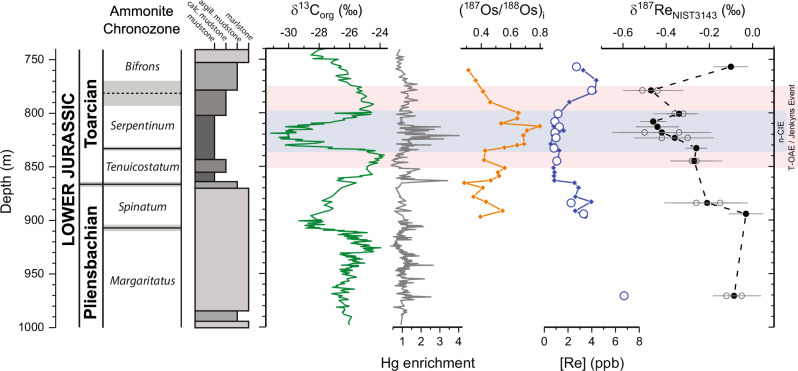
Rhenium isotopes in the Mochras core sedimentary record of the Lower Jurassic. The Toarcian Oceanic Anoxic Event (T-OAE, shaded red and the associated negative carbon isotope excursion (n-CIE) in blue) is shown with ammonite Zones alongside lithology as defined previously^55,57^. The stable carbon isotope composition of organic matter (δ^13^C_org_, green) is shown^48,53^ alongside mercury (Hg) enrichment (gray) which provides a proxy for global volcanism^10^, while the initial Os isotope ratio of seawater (^187^Os/^188^Os_i_) (orange) is sensitive to changes in continental weathering^70^. Published Re concentration data are shown by the blue line and diamond symbols^70^, with values measured in this study shown as circles. The new δ^187^Re record is shown as black circles, with duplicate measurements shown as open gray circles. Whiskers are either the 2SE uncertainties propagated from the measurements and bracketing zero delta standards or the 2 SD of duplicate or triplicate measurements, whichever is larger.

Rhenium concentrations decrease below the T-OAE negative CIE, from a value of 6.7 ng g^−1^ at 970 m depth in the Upper Pliensbachian *margaritatus* Zone to ~1 ng g^−1^ at 845 m depth, near the base of the T-OAE interval (Fig. 1). Concentrations remain at ~1 ng g^−1^ Re through the lower part of the OAE and through the negative-CIE interval, until ~800 m depth, where there is a progressive increase to ~4 ng g^−1^ Re. Decreases in Re concentrations before the main CIE have been documented in other sedimentary successions, and are thought to reflect an increase in the global proportion of anoxic seafloor, and consequent drawdown of the global seawater Re inventory prior to the T-OAE^54^.

There is notable variability in δ^187^Re values in our studied samples, from −0.03 ± 0.09‰ to −0.51 ± 0.11‰, ~4 times greater than typical analytical uncertainty (Methods, Supplementary Data 1). δ^187^Re values decrease from −0.03 ± 0.08‰ at 894.3 m depth to −0.26 ± 0.12‰ at 845.0 m in the upper Pliensbachian *spinatum* Zone, before decreasing further to a minimum value of −0.51 ± 0.11‰ within the lower Toarcian *exaratum* Subzone of the *serpentinum* Zone (at 779.2 m), coincident with the negative CIE of the T-OAE. The δ^187^Re isotope excursion into the negative CIE and during the main period of Ferrar volcanism^41^ was −0.22 ± 0.10‰, expressed as the difference between the average of measurements below the T-OAE interval (average δ^187^Re = −0.16 ± 0.08‰, *n* = 7, ±2SE, 970.8 m − 845.0 m) and within the negative CIE interval (−0.39 ± 0.07‰, *n* = 7, ±2SE, 833.0 m–808.2 m). Above the T-OAE interval, δ^187^Re values were −0.10 ± 0.08‰, a similar value as before the event.

The variability in δ^187^Re values observed at Mochras likely reflects some mixture of changes in seawater δ^187^Re values at the time of deposition, alongside potential fractionation during the burial and formation of the sedimentary deposits. We note that additional sedimentary records of δ^187^Re during the T-OAE from open marine sites are not available, leading to inherent uncertainty on the global average seawater Re isotope composition. Nevertheless, to explore the role of fractionation during sedimentary burial and diagenesis, the magnitude of Re isotope fractionation between the sediments and seawater (Δ^187^Re_sed-sw_) can be explored. An advantage of the Mochras core is that the sedimentary composition of the core remains fairly constant throughout the Lower Jurassic interval^48,53^, consisting largely of gray siltstone and silty mudstone containing ~1–3 wt.% TOC and with variable carbonate content^48,55^. Without large swings to very high TOC, it is reasonable to assume that similar Re species were buried, thus minimizing local artifacts on our putative seawater Re reconstruction from a single core site. The decoupling of our isotope and concentration data also indicate that changes in Re burial in anoxic sediments across the T-OAE^54^ was not a major driver of our record.

Deposition of the Mochras sediments occurred dominantly in open marine conditions, with sustained bioturbation^48^ and a decrease but not complete disappearance of macrofauna in the lower Toarcian suggesting the bottom waters in the Cardigan Bay Basin became oxygen depleted, but not fully anoxic^56,57^. In this scenario, soluble ReO_4_^-^ would have been scavenged from seawater and incorporated into sediments following reduction or thiolation reactions in porewaters^25,33^, with some exchange with an oxygenated water column above, meaning that sediment δ^187^Re can be fractionated from the seawater value. The only Re isotope fractionation factors determined thus far are from ab initio calculations^32^ and predict that reduced and thiolated species should be isotopically lighter than the soluble ReO_4_^-^ ion in seawater. Therefore, the Mochras core sediment δ^187^Re values are likely lower than the δ^187^Re of coeval seawater. Since there is no sedimentological evidence to suggest a parallel change in Re speciation during the T-OAE, we infer that the δ^187^Re excursion recorded in the Mochras core reflects a change in seawater δ^187^Re of a similar magnitude over this interval.

### Oxidative weathering as a driver of δ^187^Re during the T-OAE

**Fig. 2:**
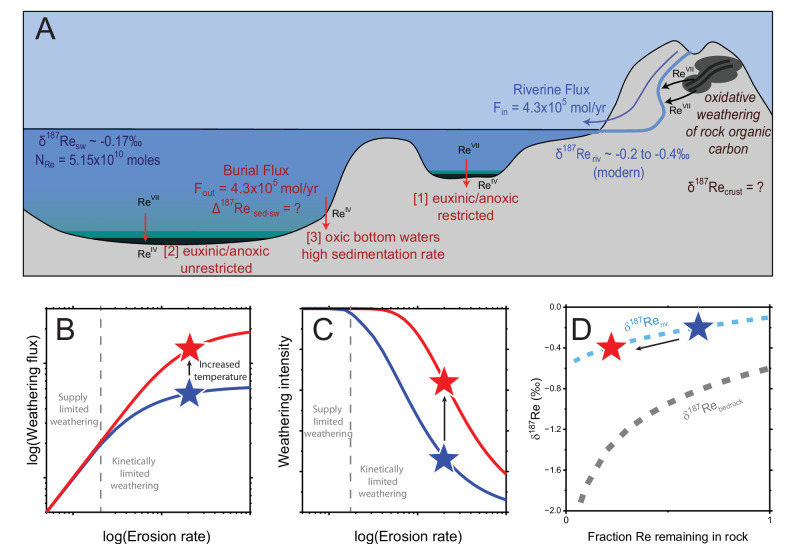
The modern rhenium cycle and the influence of weathering. **A** The major source of Re to the oceans is from rivers^25,29,34^ (Flux of rhenium into the ocean F_in_ ~ 4.3 ×10^5^ mol yr^−1^; δ^187^Re_riv_ ~ −0.2 to −0.4‰) contributing to the total oceanic Re mass (N_Re_) with seawater Re isotope composition (δ^187^Re_sw_). Re is buried in sediments under low oxygen conditions. Most Re burial occurs under anoxic bottom waters which may form in both restricted [1] and unrestricted [2] basins. In addition, Re burial can occur in settings with oxic bottom waters [3], where high sedimentation rates occur and organic matter respiration promotes the formation of anoxic pore waters, e.g., continental margins. The overall Re burial flux F_out_  ∼ 4.3 × 10^5^ mol yr^−1^, but the Re isotope fractionation factors (Δ^187^Re_sed-sw_) during burial are still unknown. The schematic figures display the effect of increasing temperature on the weathering flux (**B**) weathering intensity (**C**) and δ^187^Re_riv_ (**D**) as a function of the erosion rate, where red lines indicate warmer temperatures in the weathering zone.

In order to investigate the drivers of the negative δ^187^Re shift across the T-OAE, we developed an isotope mass balance model to reconstruct temporal variations in seawater δ^187^Re (Methods, Eqs. 2 and 5). Seawater δ^187^Re values (δ^187^Re_sw_) are controlled by the balance between the input and output fluxes to the ocean (Fig. 2)^25,29,34^. At present, Re is largely delivered to the oceans via rivers (F_riv_, 4.3 ×10^5^ mol yr^−1^) with an isotopic composition δ^187^Re_riv_ = −0.24 ± 0.10‰ (Methods). Hydrothermal vents are a net removal pathway for Re from the oceans^32,34^ with an isotopic fractionation similar in direction to Re burial in marine sediments^58^. Rhenium behaves conservatively in the oceans with concentrations of ~7.4 pg g^−1^ Re throughout the water column and a well-mixed seawater isotopic composition (δ^187^Re_sw_)^59^. We thus describe the changes in δ^187^Re_sw_ through time as a function of riverine inputs and sedimentary burial (Eqs. 2–6). The magnitude of Re isotope fractionation during burial (Δ^187^Re_sed-sw_) is a function of fluxes and fractionation factors associated with Re transformation to thiolated and reduced species, and adsorption on or co-precipitation with a range of other mineral phases (Eq. 4). We assume that the vast majority of Re burial occurs as ReO_3_S in oxygen-deficient marine sediments, with a Δ^187^Re_sed-sw_ of −0.33 ‰ (Methods). The model is applied for a duration of 0.3 Myr to provide a conservative estimate on the excursion magnitude, but durations of up to 1 Myr^40,53^ do not impact the model outcomes compared to the measured T-OAE excursion (Methods).

**Fig. 3:**
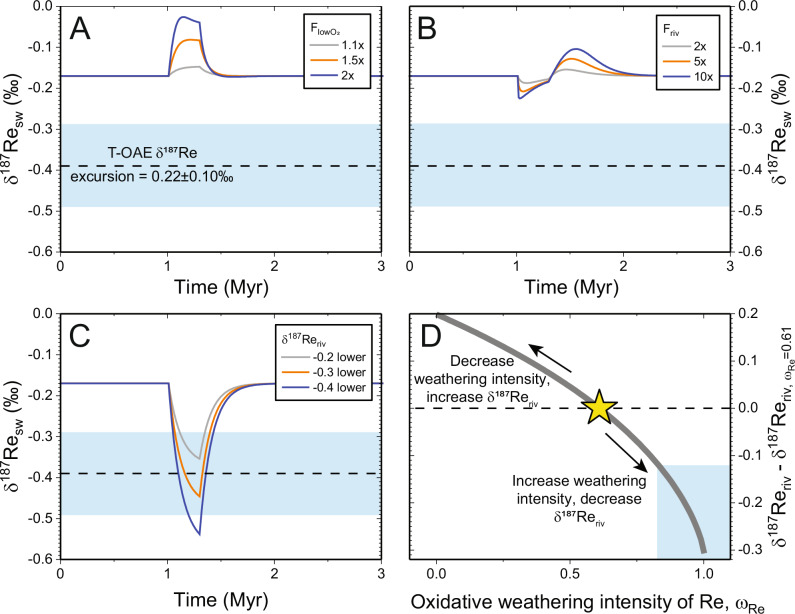
Mass balance models of the Re isotopic composition of seawater following perturbations to a steady state. Perturbations occur over 0.3 Millions of years (Myr) from 1 Myr, considering: (**A**) increasing the proportion of burial of thiolated Re species in low O_2_ sediments with Δ^187^Re_sed-sw_ values of −0.33‰; **B** increase in the riverine flux of dissolved Re; and (**C**) changing isotopic composition of river water. The dashed black line and blue shading show the magnitude of the δ^187^Re excursion into the T-OAE of −0.22 ± 0.10‰ (Fig. 1), assuming its deviation from seawater as shown in the figure. **D** Shows the modeled river δ^187^Re values (δ^187^Re_riv_) as a function of the oxidative weathering intensity of Re, ω_Re_. The star shows the ω_Re_ value of the modern day^15^, and the shift in δ^187^Re_riv_ reported relative to that initial condition. The blue box shows the range of δ^187^Re_riv_ values which would be consistent with the Toarcian Oceanic Anoxic Event (T-OAE) δ^187^Re excursion from the ocean mass balance model (**C**).

First, we explore how changes in the sedimentary Re sink from the oceans could impact δ^187^Re_sw_ through time. While the burial fractionation factors are uncertain, their sign and magnitude mean that it is unlikely that changes in the Re outputs from seawater during the T-OAE could drive a negative shift in δ^187^Re_sw_ values. The extent of global euxinia is thought to increase during the T-OAE^54^. Using a negative value of Δ^187^Re_sed-sw_ as suggested from ab initio calculations^32^, an increase in the proportion of Re buried as thiolated S species would lead to an increase in δ^187^Re_sw_. This is opposite to the shift we observe (Fig. 3A).

Having found that changes in the burial rates of Re are unlikely to explain a negative shift in δ^187^Re_sw_, we instead explore changes in the flux and isotopic composition of dissolved Re delivered to the oceans via rivers (F_riv_ and δ^187^Re_riv_) which reflect oxidative weathering processes on land. Weathering reactions are a trade-off between the rate of supply of minerals to the weathering zone, linked to erosion, versus the kinetics of the reactions which can be controlled by temperature and hydrology^17,31^. At lower erosion rates, OC_petro_ oxidation is thought to be supply limited^18,60^ with very high weathering intensity while fluxes are low. At higher erosion rates, OC_petro_ oxidation rate increases with temperature^16,19^. In these settings, OC_petro_ is supplied in excess and thus the overall weathering intensity is lower and climatically sensitive (Fig. 2B, C). These shifts in weathering intensity will impact δ^187^Re_riv_ (Fig. 2D): as weathering intensity increases, δ^187^Re_riv_ values should trend towards the value of unweathered bedrock, following more intensive oxidative weathering and near-complete Re loss^23,33^.

In the context of the T-OAE, the timescales are too short for a wholesale change in continental erosion patterns linked to tectonics which could shift OC_petro_ weathering intensity^15^. However, the increases in temperature globally^61^ should increase the intensity of OC_petro_ weathering in landscapes that are kinetically-limited^16,19^ (Fig. 2B and C). We model changes in F_riv_ from +2x to +10x and δ^187^Re_riv_ between the modern value of −0.24‰ (Methods) and a minimum value of −0.60‰ (Fig. 3B, C). A change in the riverine flux alone leads to a decrease in δ^187^Re_sw_, but this shift is small even for an unrealistically high 10-fold increase in global OC_petro_ oxidation (Fig. 3B). In contrast, a shift in δ^187^Re_riv_ of −0.24 ± 0.11‰ (to a δ^187^Re_riv_ value of −0.48 ± 0.11‰) can explain the −0.22 ± 0.10‰ shift in δ^187^Re observed in the Mochras record over the T-OAE (Fig. 3C, Methods).

Lowering of the Re isotope composition of river waters can be achieved by increasing the weathering intensity of sedimentary rock containing organic matter^23,32,33^. To determine whether these shifts are feasible in terms of predicted changes in weathering intensity of OC_petro_, we turn to a Rayleigh model to describe isotopic patterns as a function of weathering intensity^33^ (Methods, Eqs. 8 and 9). Using a range of values based on our knowledge of the modern-day Re cycle, we calculate that a decrease in δ^187^Re_riv_ values by −0.24‰ can be achieved by increasing the proportion of oxidized Re (ReO_4_^-^) leaving the weathering zone in the dissolved load of rivers from 0.61 (equivalent to modern day^15^) to 0.96 (Fig. 3D). Assuming that the single core record of δ^187^Re captures global swings in seawater chemistry during the T-OAE, for a constant rate of Re denudation, this corresponds to an increase in the global oxidized Re flux by ~1.6 times. These changes in oxidative weathering are plausible given that previous assessments have suggested a 1.6-fold^51^ to 8-fold^49^ increase in silicate weathering during the T-OAE in response to increased temperature^61^ and an enhanced hydrological cycle^48^.

### A positive feedback via OC_petro_ oxidation during warming events

Our Re isotope data indicate that there was an increase in continental oxidative weathering that peaked during the T-OAE. This increase in weathering would have released CO_2_ into the oceans and atmosphere and acted as an additional driver of global temperature change. The CO_2_ released by OC_petro_ oxidation could account for an apparent discrepancy between the CO_2_ flux from volcanism and the overall change in atmospheric *p*CO_2_ predicted by climate models. Fendley et al.^10^ used Hg concentrations in the Mochras core and Hg/CO_2_ ratios to calculate that a total of 6200 Pg C was emitted by LIP volcanism during the T-OAE interval. However, the GEOCLIM and COPSE climate models predict that additional emissions of between 8500 Pg C and 69,000 Pg C respectively are required to explain δ^13^C, *p*CO_2_ and temperature proxy data^10^.

To explore whether oxidative weathering of OC_petro_ could contribute to these “missing” CO_2_ emissions, we use recent empirical data from weathering studies to provide additional constraints on OC_petro_ weathering independent from the Re isotope record (Methods). This approach requires us to estimate: i) the apparent temperature sensitivity of OC_petro_ oxidation^16,19^; ii) the proportion of the global land surface that experiences kinetically-limited weathering^15,20^ (i.e., the supply to the weathering zone is very high); and iii) global OC_petro_ weathering flux and overall OC_petro_ weathering intensity. The apparent temperature sensitivity parameter (Q_10_, the increase in flux over a 10 °C temperature change) has been determined from modern day field based measurements in two shale weathering locations^16,19^ of 2.0 ± 0.1 and 2.7 ± 1.0. An approximate T-OAE warming^61,62^ of ~6–7 °C could thus cause a 1.6 to 2 times increase in the oxidative CO_2_ flux if weathering occurs in kinetically limited regimes (Methods).

Today, ~60% of the OC_petro_ weathering flux is attributed to highly erosive regions where oxidative weathering may be temperature sensitive (kinetically limited)^15^. Global OC_petro_ oxidation is estimated to be 68 ^+18^/_-6_ Mt C yr^−1^, with 43 Mt C yr^−1^ of incompletely weathered OC_petro_ exported to the ocean^15,20^, which equates to an average OC_petro_ weathering intensity of 0.61. Assuming that oxidative weathering on the Early Jurassic land surface was not completely supply-limited and the distribution of sedimentary rocks was not vastly different than today, we calculate that the T-OAE warming would equate to an additional 25–41 Mt C yr^−1^ release from OC_petro_ oxidation (Methods, Eqs. 12 and 13). This corresponds to an increase in global OC_petro_ weathering intensity to 0.83-0.98 if total OC_petro_ denudation remained constant. This shift in weathering intensities appears plausible based on modern observations of near-complete oxidation of OC_petro_ in slowly eroding, temperate climates^3,4,28^, alongside efficient OC_petro_ weathering in warm and humid tropical river floodplains^63^. While uncertain, these changes in global weathering flux calculated from T-OAE warming^61,62^ and an empirical temperature sensitivity of OC_petro_ oxidation^16,19^ are comparable to those suggested by modeling the decrease in δ^187^Re_riv_ values that are consistent with the measured T-OAE δ^187^Re excursion (Fig. 3D, Methods). With an approximate duration of between 0.3 and 0.5 Myr, increased OC_petro_ oxidation of 25–41 Mt C yr^−1^ would have released a total of 7600–20,490 Pg C across the T-OAE. The magnitude of this emission flux is in the range of the ‘missing’ carbon emissions that appear not to be supplied from volcanism during the event^10^.

The increase in oxidative weathering of OC_petro_ that explains the new Re isotope record and its apparent temperature sensitivity are consistent with other geochemical records. Published shifts in the Os isotope composition of seawater have been interpreted to reflect changes in silicate weathering flux^49,51^. However, sulfide minerals and OC_petro_ are enriched in Re (and ^187^Re), and other workers have suggested that enhanced oxidative weathering could increase ^187^Os supply and increase the Os isotope initial of seawater^64,65^, thus making the shifts seen in the T-OAE sedimentary record consistent with the δ^187^Re excursion (Fig. 1). An additional proxy which could reflect OC_petro_ oxidation is the stable carbon isotope record of carbonates (δ^13^C_carb_) and organic matter (δ^13^C_org_)^13,14^. In isolation, increased OC_petro_ oxidation would result in a lowering of seawater δ^13^C_carb_ and δ^13^C_org_ values as ^13^C-depleted organic matter is oxidized and supplied to the atmosphere and oceans. Unfortunately, the carbon isotope mass balance at this period of time is complicated by several factors^14,66^, most importantly: i) the unknown δ^13^C values of the volcanic inputs, particularly if rocks intrude sedimentary units^43^; ii) changes in the size of the DIC reservoir of seawater; iii) changes in δ^13^C_org_ due to changes in *p*CO_2_; iv) variation in the fluxes and proportion of carbon leaving seawater as carbonate minerals vs organic matter. Recent geological carbon cycle models have started to incorporate a dynamic organic carbon cycle and variable rates of OC_petro_ oxidation, showing these processes change our understanding of the geological regulation of the carbon cycle^67^. It is clear that future insight can come from new δ^187^Re records (Fig. 1), detrital proxies of OC_petro_ oxidation^21,22^ alongside coupled isotopic modeling of δ^187^Re, Os and C.

Our findings suggest that the weathering carbon-cycle feedbacks for the T-OAE are more complex than previously recognized. Silicate mineral weathering by carbonic acid draws down CO_2_ through increased alkalinity flux to the oceans (and when coupled to carbonate burial in the oceans) and these reactions can increase under warmer, wetter and higher *p*CO_2_ conditions, as a negative feedback. However, warmer temperatures and increased erosion under an enhanced hydrological cycle may also lead to more shale weathering and CO_2_ release by OC_petro_ oxidation. Here, we show that a negative shift in δ^187^Re values can be explained by a global increase in the weathering intensity of OC_petro_ during the T-OAE (Fig. 1), which thus acted as a positive feedback that increased the total amount of CO_2_ transferred to the ocean-atmosphere system. These conclusions support those from other major environmental perturbations, including OC_petro_ oxidation being hypothesized as an additional source of CO_2_ during hyperthermal events of the Cenozoic^21,22^. Our work suggests that oxidative weathering needs to be better accounted for to understand the complex carbon cycle interactions that operate during climatic perturbations.

## Methods

### Sample digestion

Chemical separation and Re isotope ratio measurements were conducted in the clean laboratories at the University of Oxford, Royal Holloway University of London, and Durham University following published methods^23,33,58,68^. Unless specified, all acids were distilled in either quartz or Teflon stills, and diluted with ultrapure MQ water (18.2 Ωcm resistivity).

The measurement of Re isotope ratios is challenging due to the low concentrations of Re in geological samples. To make precise and accurate Re isotope measurements, at least 1 ng Re is required, while higher Re masses allow for improved analytical precision^68^. Based on the measured Re concentrations, we aimed to digest at least 2–3 g of material for each sample. Sample powders (masses up to 500 mg) were initially digested in Savillex Teflon beakers using 3 ml 16 M HNO_3_ and 3 ml 28 M HF, on a hotplate at 160 °C for 48 h. This step digested the silicate component of the samples, and started to digest the organic matter. Samples were evaporated at 120 °C to incipient dryness and repeatedly covered in 16 M HNO_3_ and evaporated at 180 °C to destroy fluoride phases.

Samples were then transferred to Digest n-vessels purchased from QMX, specifically to break down the remaining organic matter. The vessels are composed of a digestion vessel base and collection tube lid, where acid vapors evaporating from the base vessel condense and collect. As the acid evaporates, the pressure drops in the base, allowing the acid condensing in the collection tube to fall back into the base. This process allows samples to be continually refluxed, promoting faster, more effective digestion of organic matter. 2 ml 10 M HCl and 2 ml 16 M HNO_3_ was added to the base of the vessel, and 3 ml 16 M HNO_3_ to the top collection tube. The vessels were refluxed on a hotplate at 180 °C for 48 h. After this stage, if there was no solid material left, samples were evaporated and refluxed in 5 ml 5 M HCl at 120 °C, before being finally dissolved in 20 ml 1 M HCl loading solution for column chromatography. If samples appeared sticky or cloudy at this stage, the step in the digest-n vessels was repeated to ensure complete digestion.

Two samples (SSK74550 and SSK49509) were digested using an adapted procedure to assess the approximate proportion of Re associated with the silicate versus non-silicate fraction of the sediments. Samples were initially digested using HNO_3_ and HCl in the Digest n-vessels as described above, in order to digest the non-silicate fraction. The remaining solid silicate material was separated by centrifugation and then digested using 3 ml 16 M HNO3 and 3 ml 28 M HF on a hotplate at 160 °C for 48 h. Re concentrations were measured on a Perkin Elmer NexION 5000 ICP-MS as discussed below. In these samples, only 6% of the total Re was associated with the silicate fraction, which demonstrates that there is a negligible contribution of Re from detrital silicate material. 94% of the Re was associated with the non-silicate fraction.

### Chemical separation

The procedure for Re separation from sample matrix was adapted from methods described in Dellinger et al.^68^. Biorad AG1-X8 anion exchange resin (200–400 mesh) was pre-cleaned by shaking with 5 M HCl, 8 M HNO_3_ and MQ H_2_O. 1 ml of pre-cleaned AG1-X8 resin was loaded into polypropylene Biorad columns, and cleaned with 10 ml 10 M HCl, 10 ml 1 M HCl and 30 ml 8 M HNO_3_. The resin was conditioned with 5 ml 0.5 M HNO_3_ and 5 ml 1 M HCl. Samples were loaded in ~20 ml 1 M HCl, but this volume could be increased if samples had higher mass or appeared more viscous. A further 10 ml 1 M HCl and 15 ml 0.5 HNO_3_ was used to elute the sample matrix, and then Re was collected in 16.5 ml 4 M HNO_3_. This is a higher collection volume than used by Dellinger et al.^68^, but we found this was necessary to ensure quantitative Re recovery.

The Re fractions were evaporated overnight at 100 °C, refluxed in 16 M HNO_3_ for 24 h and evaporated at 150 °C to destroy any resin particles which have bled through the column. Samples were then dissolved in 2 ml 2% HNO_3_ and a 100 µl aliquot (5%) was taken for Re concentration measurements by multi-quadrupole ICP-MS. This column procedure was repeated twice more in order to remove any remaining sample matrix. The column was conducted as described above, except samples were loaded in 1 ml 1 M HCl. Following the third column, samples were refluxed in 16 M HNO_3_ to destroy resin-derived organics, and then dissolved in 0.4 ml 3% HNO_3_ for isotopic analysis. Following three passes through the column, Re yields were typically >85%.

### Re concentration and isotope ratio measurements

Re concentrations of samples after the first pass through the anion exchange column were measured on a Perkin Elmer NexION 5000 ICP-MS at the University of Oxford. These concentration measurements were used to calculate how much sample mass was required for isotopic analysis, and to confirm that there was quantitative recovery of Re through the three column chromatography procedures. The ICP-MS was operated in standard (no gas collision cell) mode. Calibration standards of known Re concentration were run at the start of each measurement session. The typical range of calibration standards span concentrations from 1 pg g^−1^ to 100 pg g^−1^ Re. Samples were doped with 1 ng g^−1^ Rh internal standard to monitor machine drift. Certified reference materials AQUA-1 and SLRS-6 from the Canadian Geological Survey were measured throughout runs at 1x and 10x dilution to assess the accuracy of measurements. Errors are reported as the within run RSD of individual analyses, and for Re these are typically <5%.

Re isotope ratio measurements were performed following the method in Dellinger et al.^68^ using a Neptune Plus MC-ICP-MS at Durham University and Royal Holloway University of London. There are minor differences between the machine setup and running conditions, as described below.

At Durham University, measurements were made in low resolution mode, with masses ^183^W, ^184^W, ^185^Re, ^186^W, ^187^Re and ^189^Os measured on cups L3, L2, L1, C, H1 and H3 respectively. 10^13^Ω resistors were connected to cups L1 and H1 to measure the small Re beams, which are typically <100 mV. The sample introduction system consisted of a concentric flow nebulizer with flow rate of 50 µl/min coupled to a quartz cyclonic spray chamber. Typical sensitivity was ~20 mV/ppb on ^187^Re using this setup. Standards and samples were typically run at concentrations between 1 – 5 ppb, with the aim of obtaining the maximum Re signal possible for the Re concentration of that sample. Each individual measurement was composed of one block of 25 cycles with 16.7 s integration time.

The interference of ^187^Os on ^187^Re was corrected by monitoring ^189^O, and assuming a ^189^Os/^187^Os ratio of 8.219440. Mass bias and instrument drift was corrected by standard sample bracketing with the NIST 3143 standard, and doping with W, to a W/Re ratio of 20 (i.e., if the Re concentration was 5 ppb, we doped with 100 ppb W). The concentration of Re and W/Re ratio was matched within 10% for samples and bracketing standards. The correction assumes a ^186^W/^184^W ratio of 0.927672 and uses an exponential law.

At Royal Holloway University of London, the cup configuration is slightly different than that used at Durham. Measurements were made in low resolution mode, with masses ^184^W, ^185^Re, ^186^W, ^187^Re, ^188^Os and ^190^Os measured in cups L3, L2, L1, C, H1 and H2 respectively. 10^13^Ω resistors were connected to cups L2 and C to measure the small Re beams. The sample introduction system consisted of a PFA nebulizer with flow rate of 100 µl/min coupled to a quartz SIS spray chamber. Typical sensitivity was ~80 mV/ppb on ^187^Re using this setup. Standards and samples were typically run at Re concentrations of 2ppb. Each individual measurement was composed of one block of 3 cycles with 8.4 s integration of a 3% HNO_3_ blank solution, followed by one block of 40 8.4 s integrations of the sample. The average signal of the blank 3% HNO_3_ was then subtracted from the sample signal prior to carrying out the interference and mass bias corrections. The interference of ^187^Os on ^187^Re was corrected by monitoring ^188^Os, and assuming a ^188^Os/^187^Os ratio of 5.7504. Mass bias and instrument drift was corrected by standard sample bracketing with the NIST 3143 standard, and doping with W, to a W/Re ratio of 20, following the same procedure described above for Durham.

### Quality control and assurance

Re isotope ratios are reported in delta notation relative to NIST 3143 (Supplementary Data 1 and 2):

1 $$({{\rm{\delta }}}^{187}{{Re}}=({({}^{187}{{R}}{{e}}/{}^{185}{{R}}{{e}})}_{{\rm{sample}}}/{({}^{187}{{R}}{{e}}/{}^{185}{{R}}{{e}})}_{\rm{NIST\,3143}}-1)\times 1000)$$

Total procedural blanks ranged from 0.5–20 pg Re (*n* = 6), which was typically <1% of the total Re processed. The precision and accuracy of both methods was assessed by repeat analysis of the NIST 989 Re solution during measurement sequences, and analysis of USGS reference materials and other standards which had been processed alongside each batch of samples (e.g., BCR-2, SDO-1, 00N118 shale, MAG-1 and MESS 4). Over a year long period, NIST 989 had an average isotopic composition of δ^187^Re_NIST 3143_ = −0.27 ± 0.07‰ (2 SD, *n* = 16) at Durham and δ^187^Re_NIST 3143_ = −0.29 ± 0.10‰ (2 SD, *n* = 32) at Royal Holloway University of London, agreeing with previous studies (e.g., −0.28 ± 0.04‰ (2 SD, *n* = 26)^68^ and −0.27 ± 0.10‰ (2 SD, *n* = 14)^58^. Values for USGS reference materials and other in-house standards (Supplementary Data 2) agree with previous values (Supplementary Data 3).

### Rhenium isotope ocean mass balance model

We describe the evolution over time of the Re isotopic composition of seawater (δ^187^Re_sw_)^59^ as a function of the mass of Re in the seawater inventory (N_Re_, mol), the input of Re from rivers (F_riv_, mol yr^−1^) which dominates the input flux with an isotopic composition δ^187^Re_riv_, and the output of Re via sedimentary burial (F_out_, mol yr^−1^) with an isotopic composition δ^187^Re_sed_:

2 $$\frac{d({N}_{{Re}}\delta {}^{187}{Re}_{{sw}})}{{dt}}={F}_{{riv}}\times {\delta }^{187}{{Re}}_{{riv}}-{F}_{{out}}\times {\delta }^{187}{{Re}}_{{sed}}$$

Given the current state of knowledge of the Re mass balance it is convenient to describe:

3 $${\delta }^{187}{{Re}}_{{sed}}=({\Delta }^{187}{{Re}}_{{sed}-{sw}}+{\delta }^{187}{{Re}}_{{sw}})$$

where ${\Delta }^{187}{\mathrm{Re}}_{{sed}-{sw}}$ describes the fractionation factor between the sedimentary sink and seawater. This can be formulated as a mixture of a Re sink in low oxygen environments (e.g., anoxic, suboxic), and a Re sink in other sites which have higher oxygen contents^25,65^:

4 $${\Delta }^{187}{{Re}}_{{sed}-{sw}}=[(\,{f}_{{lowO}2}\times {\Delta }^{187}{{Re}}_{{lowO}2-{sw}})+({1-f}_{{lowO}2})\times {\Delta }^{187}{{Re}}_{{other}-{sw}}]$$

where ${f}_{{lowO}2}$ is the fraction of Re leaving seawater in low oxygen environments through transformation to thiolated and/or reduced species with a fractionation factor ${\Delta }^{187}{\mathrm{Re}}_{{lowO}2-{sw}}$. The removal of Re via adsorption or co-precipitation to/with a range of other mineral phases is captured in the model by ${\Delta }^{187}{\mathrm{Re}}_{{other}-{sw}}$.

We model this in incremental timesteps such that the δ^187^Re_sw_ value is calculated from the previous timestep (t-1) and the timestep duration Δt (yr) given by:

5 $${\delta }^{187}{{Re}}_{{sw}}=\frac{\left[({\delta }^{187}{{Re}}_{{sw},t-1}\times {N}_{\mathrm{Re},t-1})+({{F}_{{riv}}\times \Delta t\times \delta }^{187}{{Re}}_{{riv}})-({{F}_{{out}}\times \Delta t\times \delta }^{187}{{Re}}_{{sed}})\right]}{{N}_{{Re},t}}$$

When running perturbations to mass flux, we take account of the role of Re concentration on the output flux, and modify the output flux, F_out_* based on the perturbation above the initial Re seawater inventory, N_Re,0_

6 $${F}_{{out}}^{*}={F}_{{out}}\times \Delta t\times \frac{{N}_{{Re}}}{{N}_{{Re},0}}$$

The parameters used in the model are described herein. The scenarios were run over 5000-year timesteps and the perturbations were applied after 1 Myr for a duration of 0.3 Myr^40,53^. We use constraint on compositions from the modern Re cycle, with δ^187^Re_sw_ = −0.17 ± 0.13‰ (2 SD, *n* = 12)^59^ and a Re inventory of seawater, N_Re_ = 5.48 x 10^10^ mol. For the riverine input, only data from the Mackenzie River and its tributaries in Northern Canada has been published so far^23^, with an average δ^187^Re of −0.27 ± 0.09‰ (2 SD, *n* = 10). This is similar to the average δ^187^Re of all large global rivers measured so far (−0.24 ± 0.10‰, 2 SD, *n* = 12; Dellinger, pers. comm.) which is used as the initial δ^187^Re_riv_ value. The global pre-anthropogenic^34^ F_riv_ = 4.3 × 10^5^ mol yr^−1^ with the input of oxidized Re from rivers directly linked to oxidative weathering of OC_petro_^15,36^. F_out_ assumed to balance the inputs at mass steady state and ${f}_{{lowO}2\,}$ = 0.94 (ref. ^65^).

The isotope fractionation associated with burial of Re in the oceans remains poorly constrained by measurements, but can be determined from ab initio calculations and mass balance arguments. First, we can consider mass and isotopic steady state, which based on Eqs. 2 and 3, simplifies to:

7 $${\delta }^{187}{{Re}}_{{sw}\,}=\delta \,{{}^{187}{Re}_{{riv}}}-\Delta^{187}{{Re}}_{{sed}-{sw}}$$

For δ^187^Re_riv_ = −0.24‰ and this suggests that the overall Re isotope fractionation factor between sediments and seawater during burial (Δ^187^Re_sed-sw_) would be −0.07‰ to return the measured seawater composition given the input fluxes. Another constraint comes from ab initio calculations^32^. These suggest larger Δ^187^Re_sed-sw_ values of −0.33‰ and −0.64‰ for removal of Re as thiolated Re^VII^O_3_S^-^ and Re^VII^O_2_S_2_^-^ species respectively, with a value up to a maximum value of −1.52‰ for reduced Re^IV^Cl_6_^2-^ species^32^. In the absence of any observational or experimental evidence, we set the initial ${\Delta }^{187}{\mathrm{Re}}_{{lowO}2-{sw}}=-\!0.33{{\textperthousand}}$, which would imply a ${\Delta }^{187}{\mathrm{Re}}_{{other}-{sw}} > 0\textperthousand$ (Eq. 4). We note that the difference between seawater δ^187^Re values and modern and Holocene marine sediment reference materials (MAG-1, MESS-4) is ~−0.11‰ to −0.16‰ (Supplementary Data 2 and 3). While standard reference materials should not be used to interpret geological information, if these values were used for ${\,\Delta }^{187}{\mathrm{Re}}_{{lowO}2-{sw}}$ then ${\Delta }^{187}{\mathrm{Re}}_{{other}-{sw}} \sim 0\textperthousand$. While considerable uncertainty remains, we suggest the burial fractionation factors are likely to be towards the lower end of the theoretical values^32^. While changes in the output flux cause positive excursions and cannot explain the data (Fig. 3A), future work should prioritize quantifying these fractionation factors. We perturb the model parameters F_riv_, δ^187^Re_riv_ and ${F}_{{out}}\times {f}_{{lowO}2}$ as described in the main text and Fig. 3. We report values for a 0.3 Myr duration to provide a conservative estimate of response, given the likely range of durations from 0.3-1.0 Myr^40,53^. Longer durations will cause a slight increase in the observed excursion, but will not meaningfully impact the outcomes and interpretations in comparison to the measured δ^187^Re in the Mochras record.

### Model constraints on oxidative weathering

The oceanic Re isotopic mass balance model outputs suggest that a change in δ^187^Re_riv_ values provide the most plausible way to explain the T-OAE data (Fig. 3), with a decrease in δ^187^Re_riv_ values of −0.24 ± 0.11‰ to explain the decrease in δ^187^Re values across the T-OAE interval of −0.22 ± 0.10‰ (Fig. 3C) To provide an independent test on whether these shifts in δ^187^Re_riv_ are reasonable in terms of weathering reactions, we calculate the paired isotopic evolution of river runoff (δ^187^Re_riv_) and a weathered solid residue (δ^187^Re_w_) as a function of weathering intensity. We use a Rayleigh distillation model which has been shown to fit patterns in δ^187^Re during weathering^33^:

8 $${{\delta }^{187}{Re}}_{w}={\delta }_{0}+{\Delta }_{{riv}-w}\times {ln}({f}_{{Re}-w})$$

9 $${\delta }_{0}={{\delta }^{187}{Re}}_{w}\times {f}_{{Re}-w}+{{\delta }^{187}{Re}}_{{riv}}\times (1-{f}_{{Re}-w})$$

where δ_0_ is the initial isotopic composition of weathered rocks, f_Re-w_ is the fraction of Re remaining in the weathered residue and Δ_riv-w_ is the fractionation factor between the solution (δ^187^Re_riv_) and the residual solid (δ^187^Re_w_). The term (1 – f_Re-w_) thus describes the proportion of oxidized Re leaving the weathering zone in solution. This is a metric for the overall oxidative weathering intensity of Re, ω_Re_ = (1 – f_Re-w_) (Fig. 3D). Given the close link between OC_petro_ oxidation and Re loss during weathering of shales^15,28^, ω_Re_ is likely to be proportional to the weathering intensity of OC_petro_ (see discussion below and Eq. 11).

Given the importance of black and gray shales to OC_petro_ oxidation and Re mass balance, we take δ_0_ = −0.52‰ defined by the average of 16 shale samples from Dickson et al.^33^ and Δ_riv-w_ = 0.5‰ based on the fit to weathering profile and river data. The global average OC_petro_ weathering intensity^15,20^ is estimated to be 0.61, and if this equates to a f_Re_ = 0.39 then Rayleigh model predicts a δ^187^Re_riv_ = −0.22‰ (Eqs. 4 and 5). This is close to the published values of river δ^187^Re^23^, suggesting this approach captures the first order features of the weathering mass balance well. From the modern day initial condition, we then consider how much δ_riv_ could change from this initial condition as a function of changing f_Re_ (Fig. 3D).

### OC_petro_ weathering scenarios under warming climate

First, we consider that total OC_petro_ denudation (mass transfer, Mt Cyr^−1^) is the sum of an oxidized flux and an un-weathered flux:

10 $${J}_{{OCpetro}}={J}_{{OCpetro}-{ox}}+{J}_{{OCpetro}-{uw}}$$

Present day estimates of ${J}_{{OCpetro}-{ox}}=68$ MtC yr^−1^ and ${J}_{{OCpetro}-{uw}}=43$ MtC yr^−1^. A metric of global weathering intensity, ω_OCpetro_, can thus be described as:

11 $${\omega }_{{OCpetro}}=\frac{{J}_{{OCpetro}-{ox}}}{{J}_{{OCpetro}}}$$

We can estimate the change in J_OCpetro-ox_ during a warming event by considering that the global oxidative weathering flux and CO_2_ release is a combination of landscapes where OC_petro_ oxidation is “supply-limited”, versus those “kinetically-limited”. In areas where supply-limitation occurs, OC_petro_ oxidation is sensitive to erosion, whereas areas of kinetic-limitation describe landscapes where supply of OC_petro_ is high and temperature could influence fluxes. The kinetically-limited portion of this flux, J_KL_ is then:

12 $${J}_{{KL}}={J}_{{OCpetro}-{ox}}\times {f}_{{KL}}$$

where *f*_KL_ is the fraction of the total flux delivered by kinetically limited landscapes. Based on global analysis of OC_petro_ weathering fluxes, we take the starting point that *f*_KL_ = 0.6, reflecting the dominance of high erosion landscapes to the fluxes^15^. For a global flux of 68 MtC yr^−1^, this corresponds to J_KL_ = 41 MtC yr^−1^.

The weathering flux from kinetically limited terrains can then be modified by the apparent temperature sensitivity:

13 $${J}_{{KL}-T}={J}_{{KL}-0}\times exp \,(\alpha T)$$

where J_KL-0_ is the initial OC_petro_ weathering flux before the warming event, J_KL-T_ (Mt Cyr^−1^) is after a change in temperature, T (°C), as described by the growth rate parameter α (°C^−1^). Based on field measurements of OC_petro_ oxidation in erosive settings in France^16^ and New Zealand^19^, we define the growth parameters as between 0.069 °C^−1^ and 0.099 °C^−1^. These correspond to an apparent temperature sensitivity of OC_petro_ oxidation described by the Q_10_ parameter (increase in flux over 10 °C) of 2.0 to 2.7, respectively.

The global patterns of denudation and lithological exposure remain poorly constrained in the geological record. As such, to estimate the impact of T-OAE warming, we use the modern day OC_petro_ weathering constraints to provide an order of magnitude estimate. For 6-7 °C of warming, J_KL-0_ = 41 MtC yr^−1^ could increase by an additional 25 to 41 MtC yr^−1^. If total OC_petro_ denudation does not change, this reflects an increase in ${\omega }_{{OCpetro}}$ from an initial value of 0.61 to 0.83 to 0.98. Over the duration of a 0.3 Myr event, these fluxes suggest that OC_petro_ oxidation could have released an additional 7600–12300 PgC over the initial values. If the duration of the event was 0.5 Myr^40,53^, these totals would be 12700–20490 PgC.

These simplified calculations reflect the current state of knowledge of OC_petro_ oxidation. The magnitude of the fluxes show the need for future work to not only include OC_petro_ oxidation as a major C input, but also to include supply and kinetically-limited controls on oxidation weathering into geological carbon cycle assessments.

## Supplementary information

Description of Additional Supplementary Files

Supplementary Data 1–3

Transparent Peer Review file

## Data Availability

All rhenium isotope data generated in this study have been deposited in the UKCEH Environmental Information Data Center entry^69^ “Rhenium isotopic and geochemical characterization across lithospheric and surface processes across four global regions, 2019–2024” (10.5285/a9bc6c28-cee5-4bf9-8539-d112c0a4c3d4). The data are also provided as Supplementary Data 1–3.

## References

[1] Becker, J. A., Bickle, M. J., Galy, A. & Holland, T. J. B. Himalayan metamorphic CO_2_ fluxes: quantitative constraints from hydrothermal springs. *Earth Planet Sci. Lett.*10.1016/j.epsl.2007.10.046 (2008).

[2] Plank, T. & Manning, C. E. Subducting carbon. *Nature* **574**, 343–352 (2019). 31619791 10.1038/s41586-019-1643-z

[3] Petsch, S. T. et al. *Weathering of Organic Carbon*. in *Treatise on Geochemistry: Second Edition*10.1016/B978-0-08-095975-7.01013-5 (2013).

[4] Petsch, S. T., Berner, R. A. & Eglinton, T. I. A field study of the chemical weathering of ancient sedimentary organic matter. *Org. Geochem.*10.1016/S0146-6380(00)00014-0 (2000).

[5] Torres, M. A., West, A. J. & Li, G. Sulphide oxidation and carbonate dissolution as a source of CO_2_ over geological timescales. *Nature*10.1038/nature13030 (2014). 10.1038/nature13030 24646998

[6] Hilton, R. G. & West, A. J. Mountains, erosion and the carbon cycle. *Nat. Rev. Earth Environ.* **1**, 284–299 (2020).

[7] Walker, J. C. G., Hays, P. B. & Kasting, J. F. A negative feedback mechanism for the long-term stabilization of Earth’s surface temperature. *J. Geophys. Res.*10.1029/JC086iC10p09776 (1981).

[8] Berner, R. A. Burial of organic carbon and pyrite sulfur in the modern ocean: its geochemical and environmental significance. *Am. J. Sci.* **282**, 451–473 (1982).

[9] Berner, R. A. & Caldeira, K. The need for mass balance and feedback in the geochemical carbon cycle. *Geology*10.1130/0091-7613 (1997).

[10] Fendley, I. M. et al. Early Jurassic large igneous province carbon emissions constrained by sedimentary mercury. *Nat. Geosci.* **17**, 241–248 (2024).

[11] Clapham, M. E. & Renne, P. R. Flood basalts and mass extinctions. *Annu Rev. Earth Planet Sci.* **47**, 275–303 (2019).

[12] Herbert, T. D. et al. Tectonic degassing drove global temperature trends since 20 Ma. *Science (1979)* **377**, 116–119 (2022). 10.1126/science.abl4353 35771904

[13] Li, G. & Elderfield, H. Evolution of carbon cycle over the past 100 million years. *Geochim Cosmochim. Acta* **103**, 11–25 (2013).

[14] Hayes, J. M., Strauss, H. & Kaufman, A. J. The abundance of ^13^C in marine organic matter and isotopic fractionation in the global biogeochemical cycle of carbon during the past 800 Ma. *Chem. Geol.* **161**, 103–125 (1999).

[15] Zondervan, J. R. et al. Rock organic carbon oxidation CO_2_ release offsets silicate weathering sink. *Nature* **623**, 329–333 (2023). 37794192 10.1038/s41586-023-06581-9 PMC10632139

[16] Soulet, G. et al. Temperature control on CO_2_ emissions from the weathering of sedimentary rocks. *Nat. Geosci.* **14**, 665–671 (2021).

[17] West, A. J., Galy, A. & Bickle, M. Tectonic and climatic controls on silicate weathering. *Earth Planet Sci. Lett.*10.1016/j.epsl.2005.03.020 (2005).

[18] Hilton, R. G., Gaillardet, J. Ô, Calmels, D. & Birck, J. L. Geological respiration of a mountain belt revealed by the trace element rhenium. *Earth Planet Sci. Lett.* **403**, 27–36 (2014).

[19] Roylands, T. et al. Capturing the short-term variability of carbon dioxide emissions from sedimentary rock weathering in a remote mountainous catchment, New Zealand. *Chem. Geol.* **608**, 121024 (2022).

[20] Galy, V., Peucker-Ehrenbrink, B. & Eglinton, T. Global carbon export from the terrestrial biosphere controlled by erosion. *Nature* **521**, 204–207 (2015). 25971513 10.1038/nature14400

[21] Hollingsworth, E. H. et al. Spatial and temporal patterns in petrogenic organic carbon mobilization during the Paleocene-Eocene Thermal Maximum. *Paleoceanogr. Paleoclimatol.* **39**, e2023PA004773 (2024).

[22] Lyons, S. L. et al. Palaeocene–Eocene Thermal Maximum prolonged by fossil carbon oxidation. *Nat. Geosci.* **12**, 54–60 (2018).

[23] Dellinger, M., Hilton, R. G. & Nowell, G. M. Fractionation of rhenium isotopes in the Mackenzie River basin during oxidative weathering. *Earth Planet Sci. Lett.* **573**, 117131 (2021).

[24] Jaffe, L. A., Peucker-Ehrenbrink, B. & Petsch, S. T. Mobility of rhenium, platinum group elements and organic carbon during black shale weathering. *Earth Planet. Sci. Lett.* **198**, 339–353 (2002).

[25] Ghazi, L. et al. The global biogeochemical cycle of rhenium. *Glob. Biogeochem. Cycles* **38**, e2024GB008254 (2024).

[26] Selby, D., Creaser, R. A. & Fowler, M. G. Re-Os elemental and isotopic systematics in crude oils. *Geochim Cosmochim. Acta* **71**, 378–386 (2007).

[27] Rooney, A. D., Selby, D., Lewan, M. D., Lillis, P. G. & Houzay, J. P. Evaluating Re-Os systematics in organic-rich sedimentary rocks in response to petroleum generation using hydrous pyrolysis experiments. *Geochim Cosmochim. Acta* **77**, 275–291 (2012).

[28] Grant, K. E. et al. Validating the rhenium proxy for rock organic carbon oxidation using weathering profiles. *Chem. Geol.* **671**, 122464 (2025).

[29] Colodner, D. et al. The geochemical cycle of rhenium: a reconnaissance. *Earth Planet. Sci. Lett.* **117**, 205–221 (1993).

[30] Dalai, T. K., Singh, S. K., Trivedi, J. R. & Krishnaswami, S. Dissolved rhenium in the Yamuna River System and the Ganga in the Himalaya: Role of black shale weathering on the budgets of Re, Os, and U in rivers and CO_2_ in the atmosphere. *Geochim Cosmochim. Acta* **66**, 29–43 (2002).

[31] Hilton, R. G. et al. Concentration-discharge relationships of dissolved rhenium in alpine catchments reveal its use as a tracer of oxidative weathering. *Water Resour. Res.* **57**, e2021WR029844 (2021).

[32] Miller, C. A., Peucker-Ehrenbrink, B. & Schauble, E. A. Theoretical modeling of rhenium isotope fractionation, natural variations across a black shale weathering profile, and potential as a paleoredox proxy. *Earth Planet Sci. Lett.* **430**, 339–348 (2015).

[33] Dickson, A. J. et al. Rhenium isotopes record oxidative weathering intensity in sedimentary rocks. *Geochem. Geophys. Geosyst.* **25**, e2024GC011795 (2024).

[34] Miller, C. A., Peucker-Ehrenbrink, B., Walker, B. D. & Marcantonio, F. Re-assessing the surface cycling of molybdenum and rhenium. *Geochim Cosmochim. Acta* **75**, 7146–7179 (2011).

[35] Jenkyns, H. C. & Clayton, C. J. Black shales and carbon isotopes in pelagic sediments from the Tethyan Lower Jurassic. *Sedimentology* **33**, 87–106 (1986).

[36] Jenkyns, H. C. The early Toarcian (Jurassic) anoxic event: stratigraphic, sedimentary, and geochemical evidence. *Am. J. Sci.* **288**, 101–151 (1988).

[37] Hesselbo, S. P. & Pieńkowski, G. Stepwise atmospheric carbon-isotope excursion during the Toarcian Oceanic Anoxic Event (Early Jurassic, Polish Basin). *Earth Planet Sci. Lett.* **301**, 365–372 (2011).

[38] Kemp, D. B., Coe, A. L., Cohen, A. S. & Schwark, L. Astronomical pacing of methane release in the Early Jurassic period. *Nature* **437**, 396–399 (2005). 16163353 10.1038/nature04037

[39] Gambacorta, G., Brumsack, H. J., Jenkyns, H. C. & Erba, E. The early Toarcian Oceanic Anoxic Event (Jenkyns Event) in the Alpine-Mediterranean Tethys, North African margin, and North European epicontinental seaway. *Earth Sci. Rev.* **248**, 104636 (2024).

[40] Kemp, D. B. et al. The timing and duration of large-scale carbon release in the Early Jurassic. *Geology* **52**, 891–895 (2024).

[41] Burgess, S. D., Bowring, S. A. & Fleming, T. H. High-precision geochronology links the Ferrar large igneous province with early-Jurassic ocean anoxia and biotic crisis. *Earth Planet Sci. Lett.* **415**, 90–99 (2015).

[42] McElwain, J. C., Wade-Murphy, J. & Hesselbo, S. P. Changes in carbon dioxide during an oceanic anoxic event linked to intrusion into Gondwana coals. *Nature* **435**, 479–482 (2005). 15917805 10.1038/nature03618

[43] Heimdal, T. H., Goddéris, Y., Jones, M. T. & Svensen, H. H. Assessing the importance of thermogenic degassing from the Karoo Large Igneous Province (LIP) in driving Toarcian carbon cycle perturbations. *Nat. Commun.* **12**, 6221 (2021). 34711826 10.1038/s41467-021-26467-6 PMC8553747

[44] Hesselbo, S. P. et al. Massive dissociation of gas hydrate during a Jurassic oceanic anoxic event. *Nature* **406**, 392–395 (2000). 10935632 10.1038/35019044

[45] Pieńkowski, G., Hodbod, M. & Ullmann, C. V. Fungal decomposition of terrestrial organic matter accelerated Early Jurassic climate warming. *Sci. Rep.* **6**, 31930 (2016). 27554210 10.1038/srep31930 PMC4995404

[46] Krencker, F. N., Lindström, S. & Bodin, S. A major sea-level drop briefly precedes the Toarcian oceanic anoxic event: implication for Early Jurassic climate and carbon cycle. *Sci. Rep.* **9**, 12518 (2019). 31467345 10.1038/s41598-019-48956-x PMC6715628

[47] Izumi, K., Kemp, D. B., Itamiya, S. & Inui, M. Sedimentary evidence for enhanced hydrological cycling in response to rapid carbon release during the early Toarcian oceanic anoxic event. *Earth Planet Sci. Lett.* **481**, 162–170 (2018).

[48] Xu, W. et al. Evolution of the Toarcian (Early Jurassic) carbon-cycle and global climatic controls on local sedimentary processes (Cardigan Bay Basin, UK). *Earth Planet Sci. Lett.* **484**, 396–411 (2018).

[49] Cohen, A. S., Coe, A. K., Harding, S. M., & Schwark, L. Osmium isotope evidence for the regulation of atmospheric CO_2_ by continental weathering. *Geology* **32**, 157–160 (2004).

[50] Brazier, J. M. et al. Calcium isotope evidence for dramatic increase of continental weathering during the Toarcian oceanic anoxic event (Early Jurassic). *Earth Planet Sci. Lett.* **411**, 164–176 (2015).

[51] Percival, L. M. E. et al. Globally enhanced mercury deposition during the end-Pliensbachian extinction and Toarcian OAE: a link to the Karoo-Ferrar Large Igneous Province. *Earth Planet Sci. Lett.* **428**, 267–280 (2015).

[52] Them, T. R. et al. Evidence for rapid weathering response to climatic warming during the Toarcian Oceanic Anoxic Event. *Sci. Rep.* **7**, 5003 (2017). 28694487 10.1038/s41598-017-05307-y PMC5504049

[53] Storm, M. S. et al. Orbital pacing and secular evolution of the Early Jurassic carbon cycle. *Proc. Natl. Acad. Sci. USA* **117**, 3974–3982 (2020). 32041889 10.1073/pnas.1912094117 PMC7049106

[54] Kunert, A. & Kendall, B. Global ocean redox changes before and during the Toarcian Oceanic anoxic event. *Nat. Commun.* **14**, 815 (2023). 36781894 10.1038/s41467-023-36516-x PMC9925726

[55] Ullmann, C. V., Szűcs, D., Jiang, M., Hudson, A. J. L. & Hesselbo, S. P. Geochemistry of macrofossil, bulk rock, and secondary calcite in the Early Jurassic strata of the Llanbedr (Mochras Farm) drill core, Cardigan Bay Basin, Wales, UK. *J. Geol. Soc. Lond.* **179**, jgs2021-018 (2021).

[56] Ullmann, C. V., Thibault, N., Ruhl, M., Hesselbo, S. P. & Korte, C. Effect of a Jurassic oceanic anoxic event on belemnite ecology and evolution. *Proc. Natl. Acad. Sci. USA* **111**, 10073–10076 (2014). 24982187 10.1073/pnas.1320156111 PMC4104856

[57] Pieńkowski, G., Uchman, A., Ninard, K., Page, K. N. & Hesselbo, S. P. Early Jurassic extrinsic solar system dynamics versus intrinsic Earth processes: Toarcian sedimentation and benthic life in deep-sea contourite drift facies, Cardigan Bay Basin, UK. *Prog. Earth Planet Sci.* **11**, 18 (2024).

[58] Wang, W. et al. Rhenium elemental and isotopic variations at magmatic temperatures. *Geochem Perspect. Lett.* **28**, 48–53 (2023).

[59] Dickson, A. J., Hsieh, Y. T. & Bryan, A. The rhenium isotope composition of Atlantic Ocean seawater. *Geochim Cosmochim. Acta* **287**, 221–228 (2020).

[60] Ogrič, M. et al. Low rates of rock organic carbon oxidation and anthropogenic cycling of rhenium in a slowly denuding landscape. *Earth Surf. Process Land.* **48**, 1202–1218 (2023).

[61] Bailey, T. R., Rosenthal, Y., McArthur, J. M., van de Schootbrugge, B. & Thirlwall, M. F. Paleoceanographic changes of the late Pliensbachian-early Toarcian interval: a possible link to the genesis of an Oceanic anoxic event. *Earth Planet Sci. Lett.* **212**, 307–320 (2003).

[62] Korte, C. et al. Jurassic climate mode governed by Ocean gateway. *Nat. Commun.* **6**, 10015 (2015). 26658694 10.1038/ncomms10015 PMC4682040

[63] Dellinger, M. et al. High rates of rock organic carbon oxidation sustained as Andean sediment transits the Amazon foreland-floodplain. *Proc. Natl. Acad. Sci.* **120**, e2306343120 (2023). 37725648 10.1073/pnas.2306343120 PMC10523614

[64] Ravizza, G. & Esser, B. K. A possible link between the seawater osmium isotope record and weathering of ancient sedimentary organic matter. *Chem. Geol.* **107**, 255–258 (1993).

[65] Dubin, A. & Peucker-Ehrenbrink, B. The importance of organic-rich shales to the geochemical cycles of rhenium and osmium. *Chem. Geol.* **403**, 111–120 (2015).

[66] Derry, L. A. Closing the geologic carbon cycle. *Proc. Natl. Acad. Sci. USA* **121**, e2409333121 (2024). 39374393 10.1073/pnas.2409333121 PMC11494303

[67] Hülse, D. & Ridgwell, A. Instability in the geological regulation of Earth’s climate. *Science* **389**, eadh7730 (2025). 40997180 10.1126/science.adh7730

[68] Dellinger, M., Hilton, R. G. & Nowell, G. M. Measurements of rhenium isotopic composition in low-abundance samples. *J. Anal. Spectrom.* **35**, 377–387 (2020).

[69] Hilton, R. G. et al. Rhenium isotopic and geochemical characterisation across lithospheric and surface processes across four global regions, 2019-2024. NERC EDS Environmental Information Data Centre 10.5285/a9bc6c28-cee5-4bf9-8539-d112c0a4c3d4 (2025).

[70] Percival, L. M. E. et al. Osmium isotope evidence for two pulses of increased continental weathering linked to Early Jurassic volcanism and climate change. *Geology* **44**, 759–762 (2016).

