# Supplementary material for: Rhenium isotopes reveal enhanced rock organic carbon oxidation over the Toarcian Oceanic Anoxic Event
Source: Nat Commun. 2026 Apr 16;17:5303. doi: 10.1038/s41467-026-71533-6 (PMC13270099; doi:10.1038/s41467-026-71533-6)
Supplement: Supplementary file 1 — Description of Additional Supplementary Files [file 41467_2026_71533_MOESM1_ESM.pdf]

Description of Additional Supplementary Files:

Supplementary Data 1: Measurements of rhenium concentration and isotope composition (reported as $\delta^{187}Re_{NIST3143}$) for the Mochras Llanbedr (Mochras Farm) borehole, Cardigan Bay Basin, U.K

Supplementary Data 2: Measurements of rhenium concentration and isotope composition (reported as $\delta^{187}Re_{NIST3143}$) for standards run during this study.

Supplementary Data 3: Summary of published measurements of the rhenium isotope composition (reported as $\delta^{187}Re_{NIST3143}$) of standard materials which were run for this study (Supplementary Data 2).
