# Supplementary material for: Rhenium isotopes reveal enhanced rock organic carbon oxidation over the Toarcian Oceanic Anoxic Event
Source: Nat Commun. 2026 Apr 16;17:5303. doi: 10.1038/s41467-026-71533-6 (PMC13270099; doi:10.1038/s41467-026-71533-6)
Supplement: Supplementary file 3 — Transparent Peer Review file [file 41467_2026_71533_MOESM3_ESM.pdf]

nature portfolio

Peer Review File

Rhenium isotopes reveal enhanced rock organic carbon oxidation over the Toarcian Oceanic Anoxic Event

Corresponding Author: Professor Robert Hilton

This file contains all reviewer reports in order by version, followed by all author rebuttals in order by version.

Version 0:

Reviewer comments:

Reviewer #1

(Remarks to the Author)
Rhenium isotopes reveal enhanced rock organic carbon oxidation over the Toarcian Oceanic Anoxic Event by Stow et al.
Dear authors,
I read your manuscript carefully and found it very interesting, compelling, and full of novel ideas. The paper is well-written, the ideas are clearly explained, and the data is robust and of very high quality. Below are some comments that I believe are important to address. With my best wishes,
Abstract
• Line 18: Oxford comma after 'oceans'?
• Line 20: 'weathering is also a source' -* Wouldn't it be better to say 'weathering could also be a source'? This phrasing could help frame the paper as a proof of concept.
• Lines 30-32: Consider inverting the sentence for greater impact, especially for non-specialists: 'Oxidative weathering increased CO_2 release during the warmth of the T-OAE, demonstrating that chemical weathering does not simply act as a stabilizing feedback during past warm events.'
Introduction
• Line 49: Oxford comma after 'crustal lithology'?
• Line 58: Are these experimental conditions? If so, does the 10°C temperature increase refer to atmospheric values?
• Line 59: Consider rephrasing 'It is clear...' to something like 'Thus, it is critical to constrain...'
• Line 66: Qualitative or quantitative track?
• Lines 84-86: The negative CIE is actually superimposed on a larger positive carbon isotope excursion. Please check Reference 41 (Gambacorta et al., 2024, ESR) for a detailed discussion. Additionally, I recommend ensuring consistency in the use of 'T-OAE' throughout the manuscript.
• Lines 86-87: Consider including the most recent paper on the T-OAE duration by Kemp et al. (2024), which suggests an age of 288 Myr for the CIE (*Kemp et al., 2024. The timing and duration of large-scale carbon release in the Early Jurassic, Geology*).
• Lines 87-92: The discussion could benefit from referencing Heimdal et al. (2021) (Assessing the importance of thermogenic degassing from the Karoo Large Igneous Province (LIP) in driving Toarcian carbon cycle perturbations, Nature Communications). While not the main focus of your paper, integrating Kemp et al. (2024) would strengthen the discussion on volcanism and thermogenic degassing, given their high-precision radioisotopic ages.
Sedimentary 0^{187}Re records of the Toarcian OAE
• Line 118: (negative CIE) -* n-CIE?
• Lines 116-123: The distinction between the 'negative' and 'main' CIE is unclear. I recommend adopting the terminology proposed by Gambacorta et al. (2024) for standardization. Also, please revisit my earlier comment on 'T-OAE' usage.
• Lines 116-130: The description of [Re] using core depth and 0^{13}C stratigraphy, while 0^{187}Re variations follow ammonite zonation, is somewhat confusing. I suggest homogenizing the description under a single criterion.
Oxidative weathering as a driver of 0^{187}Re during the T-OAE
• Line 171: A shorter duration should be considered here, in line with new evidence suggesting a briefer event.
A positive feedback via OCpetro oxidation during warming events
This section would benefit from two key additions:
1. A model version incorporating the newly proposed (and more precise) duration of the T-OAE (Kemp et al., 2024).
2. A more comprehensive discussion on why the 'missing' CO_2 emissions from Fendley et al. (2024) are better explained by weathering of OC-rich rocks rather than by previously proposed mechanisms (as listed in the introduction).

Figures
Figure 1: This is a key figure, but the δ^{187}Re data—particularly the negative excursion—is difficult to discern. Consider:

- Vertically expanding the figure (e.g., by removing the Variabilis ammonite zone, where no data is present).
- Simplifying the symbols (e.g., replacing triangles with empty circles with green borders).
- Reducing visual noise by modifying error bars (e.g., removing end-tips or moving them to a supplemental figure). Additional figure notes:
- Clarify in the caption what 'mdn/sitn' means.
- Merge the light blue rectangles representing the n-CIE.
- Label the light blue rectangle (assuming it denotes the n-CIE).
- Given the exceptional quality of the Mochras record, indicate the T-OAE position (distinct from the n-CIE).
- Shouldn't ammonite zone names be in italics?

Reviewer #2

(Remarks to the Author)
In this manuscript, the authors present Rhenium geochemical data (concentration and isotopic composition) from a sedimentary rock core containing Jurassic marine sediments that span the Toarcian Anoxic Event, and use these data to drive an isotope mass balance model for Re inputs and outputs to the ocean. Enhanced oxidative weathering of OM-rich rock in the model yields results that agree with the core measurements, suggesting that during anoxic events, oxidative chemical weathering of OM may provide a source of CO2 to the atmosphere-ocean system. As such, the authors suggest that unlike silicate weathering which creates a negative feedback in the climate-hydrophere-lithosphere system, rock-derived OM weathering may form a positive feedback on the same system.
This conclusion is significant for understanding Earth's past as well as its possible future. If warming leads to a positive carbon-climate feedback due to OM weathering in addition to the well-known negative carbon-climate feedback of silicate weathering, then our projections for future atmospheric CO2 concentrations and climate states must account for this (and may suggest a slower recovery from the current pulse of anthropogenic CO2 than previously assumed).
The work builds on a base of Re geochemical analyses. The challenges and difficulty in obtaining Re isotopic compositions is to be acknowledged here, and the authors carefully describe their approach in the Methods section. As such, we can be reasonably confident that these numbers are good. However, the numbers are also few. Data are presented for twelve depths in the core; five depths have single Re isotope measurements and seven have two with calculated averages. Given the range and error of these measurements it is not entirely certain that the Re isotope data indicate a substantial decline in d187Re values during the OAE. The authors could do a stronger and more consistent job stating the magnitude of isotopic excursion that they think these data reveal; in lines 126-127 the values decrease from -0.03% to -0.26% ... before decreasing further to a minimum of -0.51%. In line 131, the authors claim a -0.5% excursion, while in line 213 it is a -0.25% excursion. The -0.51% minimum is just one of two measurements for this core depth; the other measurement is -0.44%, so it is curious why the average is not referred to here instead of the extreme.
The isotope mass balance model is intriguing, and presents a useful way to explore perturbations in the Re cycle. The authors carefully acknowledge limitation to our understanding of Re fractionation during burial under oxic and anoxic marine conditions, with the anoxic fractionation drawn only from ab initio calculations and the oxic fractionation estimated entirely from assumptions of Re steady state in the ocean. What is the value for that oxic fractionation factor? Table-S4 and the text only provide the overall fractionation factor for combined oxic and anoxic sediments. It is unclear that Re steady state in the ocean is a reasonable assumption for the deep-time past, especially across transitions into and out of widespread ocean anoxia such as the Toarcian OAE. While this does not impact how the oxic fractionation factor was estimated (as this is based on the modern ocean and its inputs), the subsequent model sensitivity analyses do rest on an assumption of steady state Re mass in the oceans. The modern inputs and outputs listed on Table-S4 suggest a residence time for Re in the ocean of ~1.2 Myr, which is not long enough to assume that changes in Re outputs could not drive changes in Re concentration in the ocean. While the authors do allow for changes in Re burial in anoxic sediments in their model (e.g. Figures 2 and 3), total Re burial is not independent of river Re input but instead is matched to this to maintain ocean steady state. There may be a slight error in Equation 1 (line 164); should the first term after the equals sign be Friv X d187Re-river? Setting aside concerns about magnitude of Re isotopic excursion and Re steady state, the crux of the authors' argument is that a negative Re isotopic excursion on this OAE timescale likely reflects a negative shift in the 187Re value of river inputs. The authors reasonably assign this shift to an increase in weathering of OM-rich rocks. One can assume that within the Monte Carlo simulations there are some that combine the increased river flux of Figure 3A with the negative shift in river Re isotope values of Figure 3B as these two forcings should act in tandem. As such, I wonder if the authors could estimate the mass of CO2 release from the modeled change in river Re flux (and assumptions about Re/C ratios) in addition to the CO2 estimate from temperature sensitivity of oxidative weathering fluxes. The assertion that this all represents a feedback is intriguing, and hopefully will be supported by further study that included mechanistic model of Re mass and isotopic systematics during OM weathering.
There appears to be a typo in line 229.
It would be useful if in Figure 3 the authors not only highlighted the average of the Monte Carlo simulations but also described those simulations that best align with a -0.25% isotope excursion.
It is not clear what Figure 2 panels B, C and D bring to the paper beyond what is described in the text.

Reviewer #3

(Remarks to the Author)
Introduction:

This is a well-written and well-argued manuscript, with state-of-the-art isotope data (though see my comments on the Os correction) and good documentation of analytical results, including those of geochemical reference materials. The topic is of broad interest and deals with an important issue in geochemistry and Earth's past climate. It is thus potentially of interest to the broad Nature Communications community.

Arguments:

The key argument is that isotope fractionation of rhenium during weathering of lithologies rich in organic carbon initially release isotopically heavy Re to rivers and the ocean. The more complete weathering is, the more the isotopic composition mimics that of the bulk lithology. This bulk composition is likely isotopically lighter than ambient seawater. Events in earth history that approach complete Re (and related organic carbon) release, such as brief hyperthermals, should therefore cause a negative excursion in the Re isotope record of seawater. This is what the authors claim to have discovered during the Toarcian OAE (TOAE).

Model:

The authors then build a quantitative model to explore various transient scenarios. These, for my taste, could be improved. For instance, I urge the authors to develop a coupled Re-Os-C isotope model to quantitatively test which of their scenarios match the observations best. For instance, Dubin and Peucker-Ehrenbrink (2015) have estimated the average Os isotope composition of the types of shales the authors investigated for Re. Together with other literature data, average Re-Os-C concentrations relationships can be established. These isotope tracers can then be coupled to data on the release from weathering sections of black/gray shales. If a model can be devised that matches the observed Re, Os and C isotope data for the TOAE, the study merits publication in Nature Communications. For instance, does the observed increase in 187Os/188Os during the TOAE match the predicted decrease in Re isotope composition and the release of the "missing" C from OCpetro, if an 187Os/188Os of ~2 is used for extra release from organic-rich shales during this event? If not, what isotope composition would result in a match. What does the predicted δ13C tell us about the additional OC source? Analogous questions can be asked for carbon and its isotopes. Such a refined/expanded model will have to deal with the change in residence time that is indicated by the low Re concentrations in sediments deposited during the TOAE (lines 121132), and related effects on the response of the system to perturbation (a la Lasaga & Berner, 1998). It would help the readers to have the data for the TOAE section plotted on the model results (Fig. 3) in order to be able to see how well the observations match the model.

I have to admit that I am skeptical of the "weathering intensity" argument. Weathering of gray/black shale lithologies and related release of Re and C is quick and fairly complete, aided by acidic conditions created by the quick oxidation of sedimentary pyrite. I therefore have difficulties accepting the notion of incomplete weathering of such lithologies under almost any near-surface conditions. For instance, will a 10 degree C change in temperature really matter? I know experiments indicate this (under idealized conditions), and evidence from silicate weathering also indicates temperature dependence. I do think these OM-rich lithologies are special and the matter associated with the organic matter is released quickly and nearly completely. That means that Re that is released during weathering will always mostly reflect the isotopic composition of the source lithology, which will (with fractionation) be linked to the contemporaneous isotope composition of seawater which likely will have varied through time. Such a Re isotope record of paleo-seawater still needs to be reconstructed. At the moment we know little about the Re isotopic composition of continental runoff (and SGD) as only values for the Mackenzie River have been published. To me, it is a global flux issues that is related to the intensity of the hydrologic cycle, rather than a "weathering intensity" issue. I could be convinced otherwise if – for instance – the CIA (or other quantitative measures of "weathering intensity") in the detrital fraction changed during the TOAE. Has it?

There is an additional complication – the role of hydrothermal circulation as an additional player in the global cycle. The authors do not correctly characterize the findings of Miller et al. (2011) regarding submarine HT hydrothermal systems (line 160). These systems are a Re sink, NOT a small source. Miller et al. write that Re is more quickly lost from fluids than Mg, an element that – at zero concentration (i.e. complete removal from seawater) – is used to characterize hydrothermal end-members. Relevant removal fluxes have been calculated by Ghazi et al. (2024, full disclosure – I am a coauthor of that study). To my knowledge submarine LT hydrothermal systems have not yet been investigated for Re isotope composition and concentration.

Additional issues:

Line 35: I don't think it's quite correct to state that "Earth's climate is controlled by variations in the concentration of atmospheric CO2". Orbital parameters (Milankovic) and global albedo matter as well, among others.

Lines 75/76: "caused by the preferential oxidative weathering of phases enriched in the heavy 187Re isotope" is an interesting characterization as it implies that there are phases in these shales that are enriched in 187Re, and that those phases release their Re first. I don't think of the system in this way because I think isotope fractionation, differential reaction energies and the stability of molecules transporting either 185Re or 187Re are the key. A study of the Re isotope composition extractable organic phases in such shales could provide an answer.

Lines 104/5: I think Ravizza et al. (2000) provided the best empirical evidence for weathering feedback operating in the geologic past (in their case the PETM). I also think the link between the Re-Os system and the marine (organic) carbon record, explored by Ravizza & Esser (1993), was very farsighted.

References: many subscripts are missing.

Methods:

Line 470/4: I wonder why the authors did not ash the samples prior to dissolving them in mineral acids. This should have made the whole digestion process much less onerous.

Line 523 and some data tables: it would be better to replace “ppt” and “ppb” with “pg/g” and “ng/g” to avoid confusion with volumetric measures (parts per... what parts? Weight or volume?)

Line 542: I do not understand why the authors use a fixed 189Os/187Os ratio for the interference correction, and if they use one I don’t understand why they choose a nearly chondritic ratio. These are presumable radiogenic gray shales which would require a lower 189Os/187Os ratio that 8.21944 (the 188Os/187Os value of 5.7504 used at RHUL is a little better, but still much too close to chondritic – a value of ~1.6 would be better). Ideally, the authors used the Os isotope values measured for these samples. I assume these details do not matter in practice because the interferences are likely small, but it would look cleaner if these details were taken into account. How large are the 188Os and 189Os ion beams and the related correction of the 187Re signal?

Recommendation: If the authors revise the manuscript and make the model more quantitative it may be suitable for Nature Communications. I’d be happy to review such a revised version.

I don’t like anonymous reviews. Please do not hide my identity from the authors.
Bernhard Peucker-Ehrenbrink (WHOI)

Version 1:

Reviewer comments:

Reviewer #1

(Remarks to the Author)
Dear authors,
I have read the revised version of your research article, and I appreciated the time you took to address my comments. In my opinion, the paper is suitable for publication.
However, I have identified some minor details that might improve the final publication that I pointed below.
Again, with my best wishes,
MR

Line 21: ‘shales’
Lines 23-25: consider to include ‘Early’ to ‘Jurassic’ in lines 23 and remove ‘Early Jurassic’ in lines 25 to avoid saying Jurassic twice in the sentence.
Line 41: changeS?
Line 93: remove ‘was’
Line 123: ‘decrease at a level’ is ok?
Line 138: the ‘Ferrar’ volcanism?
Figure 1: ammonite zones are still not italicized
Try to center LOWER JURASSIC (in the present version it is slightly inclined to the top.
Figure 2: consider making the markers (in B, C, D) longer for a better visualization. Similar in figure 3.
Line 333: consider adding spaced between -0.22 and the plus minus symbol.
Methods: please, check carefully how do you described the amount of volume you used. In some case it is reported as ‘1ml’ and some as ‘1 ml’, meaning you alternative separate the number from the unit. Please, make sure that it is consistent in one way throughout the manuscript.

Line 633: I think the abbreviation of RHUL here is unnecessary. It likely should be before.

In the manuscript in general, pCO2 should’t be pCO2?

Reviewer #2

(Remarks to the Author)
The revisions that these authors completed have further strengthened the manuscript. I am glad to see that my suggestion to explore the mass of CO2 release led the authors to expand their modeling effort, especially around weathering intensity and

C fluxes (I was reviewer #2 of the original manuscript). My only remaining comment is that it is initially unclear that the x-axis in Panel B in revised Figure 3 is time based on the way that the four panel figures are arranged.

Reviewer #3

(Remarks to the Author)
2nd review of Stow et al. “Rhenium isotopes reveal enhanced rock organic carbon oxidation over the Toarcian Oceanic Anoxic Event”, submitted to Nature – Reviewer: Bernhard Peucker-Ehrenbrink (WHOI)

The authors argue that the observed ~0.22 per mil decrease in the stable Re isotope composition of seawater that coincides with the Toarcian Ocean Anoxic Event (T-OAE) negative carbon isotope excursion was caused by enhanced delivery of rhenium liberated by more intense oxidative weathering of petrogenic organic carbon due to enhanced global temperatures/enhanced hydrologic cycle. It is valuable to recall that this claim is based on 12 measurements on a single sediment core that is supposed to represent the global ocean. Nature portfolio journals are supposed to present provocative ideas and this manuscript qualifies, in my view. Personally, it would make me more comfortable to have two sediment records from separate ocean basins as a basis for this story/model; but then, I’m not the author. I think this is an editorial decision.

Overall, I do appreciate the way the authors dealt with reviewers’ feedback on the original manuscript. I like the use of present-day petrogenic OC weathering intensity (~0.6, based on river export assuming steady state) to place the model results for the T-OAE event (0.83-0.98) in a modern-day context. The model requires the equivalent of a 1.6-fold increase in the global Re flux to seawater. Increasing the weathering intensity to ~0.9 means that very little petrogenic Re (or OC) was transported in the riverine particulate load during this event. This, of course, would mean that liberated OC is either part of the DOC load or is converted to CO2 during oxidation/transport, thus helping to balance the T-OAE carbon imbalance. Still, I wonder how realistic such extreme weathering intensities are. I do like that the authors also consider the implications of such a scenario for the marine Os isotope record (lines 267-273), as all three cycles (C, Re, Os) are coupled, to some extent.

I do wonder why the authors cling to the notion that hydrothermal vents are a minor source of Re (line 173). While there is Re coming out of such vents, nobody would claim – by analogy – that such vents are a source of sulfur (sulfate) to seawater despite the fact that there is often measurable sulfate in vent fluids. Or – a human analog – we would not claim that humans are a source of oxygen to the atmosphere just because the air we exhale contains some oxygen. As far as we know submarine hydrothermal circulation acts as a marine sink of Re, and there are indications that it is removed more quickly than sulfate from seawater, an element that is used (at zero concentration) to define the hydrothermal end-member (by extrapolation of linear concentration trends). Interestingly, this sink (or source in the authors’ view) is not shown in Fig. 2.

Minor points:

Following USGS nomenclature, I suggest the authors use “y” or “yr” to denote duration of geologic events, not “a” (reserved for time points in the past, i.e. years “ago”).

I also recommend to consistently separate values and related units by a space (e.g. 10 ml instead of 10ml, or 6 M instead of 6M, or Mt C instead of MtC). It would also be better to use gram-based units (ng/g) instead of “parts per xxx” (ppb) that are ambiguous (parts of what, weight or volume?)

It would be better to switch to “radiogenic and stable” in line 100 to refer to “osmium and carbon” (line 101).

Is it “Digest-n vessels” (line 559) or “Digest n-vessels” (line 573-574)?

I wonder why the authors do not correct the way the data are corrected for contributions from 187Os on 187Re via 189Os (lines 627-628, 644) – I know, it’s something for sticklers, but it would show that the authors care about such analytical details. It’s just a single value that needs to be changed for each sample in the code and then this issue is gone. It won’t change the results because the effect is so small, and therefore would require minimal changes.

There is a reference number (REF) missing in line 686.

Line 688: “Mackenzie River”, not “Mackenzie river” (it’s a name).

Line 700, no blank space between “and” and the following comma.

The supplemental data table S3 currently has no values for “n” (number of analyses) for the 2009 Miller dataset. Christian Miller’s PhD thesis in the MIT-WHOI JP lists this number as 22 (Table 3.3, page 149). The electronic version of this thesis is freely available through the MIT website. This value (n=22) is also shown in the Miller et al. (2009) publication (Tab 3, page 1073).

Summary:

Overall, I think this is a much improved version that fits the type of provocative manuscripts that are of interest to the broader Earth Sciences community.

Open Access This Peer Review File is licensed under a Creative Commons Attribution 4.0 International License, which permits use, sharing, adaptation, distribution and reproduction in any medium or format, as long as you give appropriate credit to the original author(s) and the source, provide a link to the Creative Commons license, and indicate if changes were made.
In cases where reviewers are anonymous, credit should be given to 'Anonymous Referee' and the source.
The images or other third party material in this Peer Review File are included in the article's Creative Commons license, unless indicated otherwise in a credit line to the material. If material is not included in the article's Creative Commons license and your intended use is not permitted by statutory regulation or exceeds the permitted use, you will need to obtain permission directly from the copyright holder.
To view a copy of this license, visit https://creativecommons.org/licenses/by/4.0/

Reply to reviews "Rhenium isotopes reveal enhanced rock organic carbon oxidation over the Toarcian Oceanic Anoxic Event"

We have worked to addressed the concerns with significant additional work and we are confident we have comprehensively addressed the comments. Our full point by point reply is contained herein but, in summary here, the major changes we have made are:

1) A new model where we predict changes in river 0^{187}Re values for a given change in oxidative weathering intensity (Figure 3D). This is used with outputs of our oceanic Re mass balance model to assess bounds on predicted changes in weathering intensity. This analysis is then compared to the independent modelling of CO_2 emissions model based on apparent temperature sensitivity of OC_{petro} oxidation. The range of weathering intensity values and predicted shifts in weathering fluxes are consistent. Together these provide robust support to our main findings.
2) Edited the text to better explain the current state of knowledge about OC_{petro} oxidation and global estimates of weathering intensity to clarify some misconceptions.
3) New text which describes how the new 0^{187}Re data is consistent with other isotope proxies which could reflect OC_{petro} weathering, including Osmium isotopes and the stable C isotope mass balance. These revisions also point out new papers which have attempted to include OC_{petro} oxidation, but still lack any climate and denudation forcing of associated CO_2 emissions, and we summarise a need to move forward in the coupled modelling of these processes.
4) A revision of how we calculate the δ^{187}Re T-OAE "excursion" and refer to it in the manuscript. We now take the average of 7 measurements before the event (depths 970.8 m to 845.0 m) and compare that average and 2 x standard error to the average within the negative carbon isotope excursion of the T-OAE (833.0 m– 808. 2 m). This gives a δ^{187}Re shift of -0.22 ± 0.10‰, which is now referred to across the manuscript, including in the abstract. This value is also used to constrain the models whose parameters match this range of values.
5) Changed key aspects of the model set up – including variation in the duration of the model from 0.5 Ma to 0.3 Ma, the minimum current estimate, which provides a conservative estimate of excursion impact, while not impacting the resultant conclusions drawn from the modelling framework.
6) Made major revisions to the methods to include the full description of the model and its parameters and how they were quantified.
7) Fully modified Figures 1 and 3 in response to these changes and the reviewers suggestions.

Reviewer #1

Rhenium isotopes reveal enhanced rock organic carbon oxidation over the Toarcian Oceanic Anoxic Event by Stow et al.

Dear authors,

I read your manuscript carefully and found it very interesting, compelling, and full of novel ideas. The paper is well-written, the ideas are clearly explained, and the data is robust and of very high quality. Below are some comments that I believe are important to address. With my best wishes,

Thank you for these positive comments and the detailed review. We reply

below. Abstract

- Line 18: Oxford comma after ‘oceans’?

Fixed.

- Line 20: ‘weathering is also a source’ → Wouldn’t it be better to say ‘weathering could also be a source’? This phrasing could help frame the paper as a proof of concept.

Modified.

- Lines 30-32: Consider inverting the sentence for greater impact, especially for non-specialists: ‘Oxidative weathering increased CO_2 release during the warmth of the T-OAE, demonstrating that chemical weathering does not simply act as a stabilizing feedback during past warm events.’

Thanks, edits made.

Introduction

- Line 49: Oxford comma after ‘crustal lithology’?

Fixed.

- Line 58: Are these experimental conditions? If so, does the 10°C temperature increase refer to atmospheric values?

The two papers cited are from modern, field-based measurements and so this refers to atmospheric temperature rise – we have edited to clarify.

- Line 59: Consider rephrasing ‘It is clear...’ to something like ‘Thus, it is critical to constrain...’

Agreed – modified as suggested.

- Line 66: Qualitative or quantitative track?

Edited for clarity - “could provide quantitative insight on oxidative weathering”

- Lines 84-86: The negative CIE is actually superimposed on a larger positive carbon isotope excursion. Please check Reference 41 (Gambacorta et al., 2024, ESR) for a detailed discussion. Additionally, I recommend ensuring consistency in the use of ‘T-OAE’ throughout the manuscript.

We have clarified this important detail about the event in the text, thank you.

We interpret the second comment to refer to whether we are referring to the negative CIE during the T-OAE and have checked we have been consistent in the revised version. We have also edited Figure 1 to make clear these differences.

- Lines 86-87: Consider including the most recent paper on the T-OAE duration by Kemp et al. (2024), which suggests an age of 288 Myr for the CIE (*Kemp et al., 2024. The timing and duration of large-scale carbon release in the Early Jurassic, Geology*).

Thank you for highlighting this recent paper which we already cite at this point in the manuscript. We are somewhat confused by the suggestion, however, because we already cite the key findings of that work in this paragraph.

Importantly, the new evidence for duration of the T-OAE presented by Kemp et al. (2024) is intriguing, but by no means definitive. Other studies have indicated longer durations for the T-OAE. For example, cyclostratigraphic study of the Mochras core (Pienkowski et al. 2024, Progress in Earth and Planetary Science 11:18 https://doi.org/10.1186/s40645-024-00612-3) indicates a duration of at least 800 kyr, much more similar to previous estimates (see Hesselbo et al. 2020 [i.e. Jurassic chapter GTS2020] for further discussion). The short duration advocated by Kemp et al. (2024) would result in highly anomalous sedimentation rate increases for the T-OAE interval in many sections around the world, not least in Mochras. Whilst we cannot definitively rule out a short duration for the T-OAE, it is sensible to consider it a lower bound of a range of durations.

- Lines 87-92: The discussion could benefit from referencing Heimdal et al. (2021) (Assessing the importance of thermogenic degassing from the Karoo Large Igneous Province (LIP) in driving Toarcian carbon cycle perturbations, Nature Communications). While not the main focus of your paper, integrating Kemp et al. (2024) would strengthen the discussion on volcanism and thermogenic degassing, given their high-precision radioisotopic ages.

We agree that this is not the main focus of our paper, and there has been considerable prior discussion regarding the role of the Karoo LIP as the ultimate initial driver (which we do not challenge here). However, we agree that the Heimdal reference is appropriate and useful here, so have added it to the discussion.

Sedimentary δ^{187}Re records of the Toarcian OAE

- Line 118: (negative CIE) → n-CIE?

While this could save some space and words, we have decided to keep “negative” here.

- Lines 116-123: The distinction between the ‘negative’ and ‘main’ CIE is unclear. I recommend adopting the terminology proposed by Gambacorta et al. (2024) for standardization. Also, please revisit my earlier comment on ‘T-OAE’ usage.

Apologies that this was not clear – we have revised the text here and checked through the manuscript to ensure clarity. The updated Figure 1 should also now make this clear.

- Lines 116-130: The description of [Re] using core depth and δ^{13}C stratigraphy, while δ^{187}Re variations follow ammonite zonation, is somewhat confusing. I suggest homogenizing the description under a single criterion.

We have not made any edits here because the [Re] data is discussed only briefly, and to point out agreement with published data.

The following paragraph reports the novel δ^{187}Re data uses the ammonite zonation to ensure workers familiar with these excursions can follow at this point. However, we agree that adding the depths here will allow easier comparisons so have made those edits.

Oxidative weathering as a driver of δ^{187}Re during the T-OAE

- Line 171: A shorter duration should be considered here, in line with new evidence suggesting a briefer event.

Thank you. Yes, we have re-run the model to use the lower bound duration age of 0.3 Ma, taking into account recent papers on this topic (including Kemp et al., 2024), and recognising the event could have been as long as 1 Ma. This does not cause any material change to the main results or discussion.

A positive feedback via OCpetro oxidation during warming events

This section would benefit from two key additions:

1. A model version incorporating the newly proposed (and more precise) duration of the T-OAE (Kemp et al., 2024).

We have made this change – see above. Changing the timescales shown in Figure 3 (rhenium isotope model) does not cause a major material change to the manuscript or conclusions.

2. A more comprehensive discussion on why the ‘missing’ CO_2 emissions from Fendley et al. (2024) are better explained by weathering of OC-rich rocks rather than by previously proposed mechanisms (as listed in the introduction).

We have done this by completely rewriting the final section in response to this comment and those by the other reviewers. We now include more discussion on this theme. However, it is important to note that Fendley et al. (2024) do not actually discuss in detail a set of mechanisms to explain the “missing CO_2” that they cannot account for through the LIP

volcanism (and any associated thermogenic sources). Instead, they suggest "*The discrepancies between our Hg-based estimates and the two carbon-cycle model scenarios suggest that our understanding of the early Jurassic carbon cycle is incomplete. Recent studies show that slow, state-dependent, positive-feedback processes leading to high Earth system sensitivity*" before citing a set of studies based on modern processes and other past climatic change (e.g. Miocene) and including a reference to oxidative weathering of sedimentary rocks in the modern day (which is what our work now tackles). As such, we have not discussed these other proposals, namely because they were not actually identified for the T-OAE by the Fendley et al., 2024, paper.

To our best knowledge, only changes in OC_{petro} weathering would impact Re isotopes in global seawater in the way the data show. As such, we are left with only OC_{petro} weathering in rocks as a viable mechanism to explain the isotope data we have. And using those shifts and the model framework we introduce, we can explain an additional CO_2 source through this process. This discussion is now included in the revised manuscript.

Figures

Figure 1: This is a key figure, but the δ^{187}Re data—particularly the negative excursion—is difficult to discern. Consider:

We agree the clarity of this figure is of upmost importance. We have redrafted it to make the new data clear alongside the published datasets.

- Vertically expanding the figure (e.g., by removing the Variabilis ammonite zone, where no data is present).

This is a good suggestion and we have edited the figure to expand the section.

- Simplifying the symbols (e.g., replacing triangles with empty circles with green borders).

We have simplified the symbology.

- Reducing visual noise by modifying error bars (e.g., removing end-tips or moving them to a supplemental figure).

We note that because Re isotopes remain a novel and new dataset, we have kept the uncertainties on the plots, but the drafting is hopefully clearer now.

Additional figure notes:

- Clarify in the caption what 'mdn/sitn' means.

This referred to lithology but was not defined. The log has been redesigned and the lithologies now given in full.

- Merge the light blue rectangles representing the n-CIE.

Done.

- Label the light blue rectangle (assuming it denotes the n-CIE).

Done.

- Given the exceptional quality of the Mochras record, indicate the T-OAE position (distinct from the n-CIE).

Done.

- Shouldn't ammonite zone names be in italics?

Fixed.

Reviewer #2

In this manuscript, the authors present Rhenium geochemical data (concentration and isotopic composition) from a sedimentary rock core containing Jurassic marine sediments that span the Toarcian Anoxic Event, and use these data to drive an isotope mass balance model for Re inputs and outputs to the ocean. Enhanced oxidative weathering of OM-rich rock in the model yields results that agree with the core measurements, suggesting that during anoxic events, oxidative chemical weathering of OM may provide a source of CO_2 to the atmosphere-ocean system. As such, the authors suggest that unlike silicate weathering which creates a negative feedback in the climate-hydrophere-lithosphere system, rock-derived OM weathering may form a positive feedback on the same system.

This conclusion is significant for understanding Earth's past as well as its possible future. If warming leads to a positive carbon-climate feedback due to OM weathering in addition to the well-known negative carbon-climate feedback of silicate weathering, then our projections for future atmospheric CO_2 concentrations and climate states must account for this (and may suggest a slower recovery from the current pulse of anthropogenic CO_2 than previously assumed).

Thank you for this summary and positive comments on the importance of our study. We are glad the main messages were easy to follow.

The work builds on a base of Re geochemical analyses. The challenges and difficulty in obtaining Re isotopic compositions is to be acknowledged here, and the authors carefully describe their approach in the Methods section. As such, we can be reasonably confident that these numbers are good. However, the numbers are also few. Data are presented for twelve depths in the core; five depths have single Re isotope measurements and seven have two with calculated averages. Given the range and error of these measurements it is not entirely certain that the Re isotope data indicate a substantial decline in d187Re values during the OAE. The authors could do a stronger and more consistent job stating the magnitude of isotopic excursion that they think these data reveal; in lines 126-127 the values decrease from -0.03‰ to -0.26‰ ... before decreasing further to a minimum of -0.51‰. In line 131, the authors claim a -0.5‰ excursion, while in line 213 it is a -0.25‰ excursion. The -0.51‰ minimum is just one of two measurements for this core depth; the other measurement is -0.44‰, so it is curious why the average is not referred to here instead of the extreme.

We agree that the rhenium isotope system is analytically challenging and we thank the referee for recognising that is why we do not have as many measurements as for other isotope systems (although we note that there are only 25 published osmium isotope initial measurements for this period, and we have made 12 rhenium isotope measurements, 7 of these in duplicate).

We apologise that the reference to the data was not as clear as it should have been. Based on this and the comments from the editor about a more rigorous treatment of the data, we now report the difference between different parts of the core record systematically, using all the measured values and their average and twice standard error. All data are provided in Table S1.

This **major revision is captured in this section of new text**:

"The δ^{187}Re isotope excursion into the negative CIE and main period of volcanism was -0.22 ± 0.10‰, expressed as the difference between the average of measurements below (average δ^{187}Re = -0.16 ± 0.08‰, n = 7, ±2SE, 970.8 m - 845.0 m) and within (-0.39 ± 0.07‰, n = 7, ±2SE, 833.0 m – 808.2 m) the negative CIE excursion.".

We then refer to this value of -0.22 ± 0.10‰ throughout the manuscript and use it in the modelling framework.

The isotope mass balance model is intriguing, and presents a useful way to explore perturbations in the Re cycle. The authors carefully acknowledge limitation to our understanding of Re fractionation during burial under oxic and anoxic marine conditions, with the anoxic fractionation drawn only from ab initio calculations and the oxic fractionation estimated entirely from assumptions of Re steady state in the ocean. What is the value for that oxic fractionation factor? Table-S4 and the text only provide the overall fractionation factor for combined oxic and anoxic sediments.

The reviewer is correct that the oxic fractionation factor is derived based on the steady-state solution to produce an ocean with δ^{187}Re value = -0.17‰ in the model. We are glad this was clear. We note that the oxic removal of Re is thought to be minor based on work we cite, and is 6% of the burial flux in the model, with the remaining 94% suboxic/anoxic.

As discussed in the methods "Rhenium mass balance model", for the mass of Re present in the modern ocean (7.12 ppt), a river input value of $\delta_{187}Re_{sw}$ = -0.24‰, a seawater value of $\delta_{187}Re_{sw}$ = -0.17‰, and an oxic removal pathway that is either too small to have an effect on the SW mass balance or is isotopically indistinct, the SW-sediment difference of an assumed Re-S burial phase must be in the region of ~ -0.07 ‰.

However, this value should be viewed, at present, as a tuneable parameter of the model and not a meaningful, interpretable variable. For example, if the suboxic/anoxic $\Delta^{187}Re_{sed\text{-}sw}$ value was smaller (e.g. more like the -0.11‰ implied by the standard reference materials), the value of oxic $\Delta^{187}Re_{ed\text{-}sw}$ would be ~0 ‰ in the model to return a $\delta_{187}Re_{sw}$ = -0.17‰.

We have now added all the underlying equations in the model in the manuscript methods section. We have also added text which discusses these features in the methods section and provides all the relevant values in the model. It is clear that measurements of the oxic, suboxic and anoxic removal of Re from seawater are a future research priority.

It is unclear that Re steady state in the ocean is a reasonable assumption for the deep-time past, especially across transitions into and out of widespread ocean anoxia such as the Toarcian OAE. While this does not impact how the oxic fractionation factor was estimated (as this is based on the modern ocean and its inputs), the subsequent model sensitivity analyses do rest on an assumption of steady state Re mass in the oceans. The modern inputs and outputs listed on Table-S4 suggest a residence time for Re in the ocean of ~1.2 Myr, which is not long enough to assume that changes in Re outputs could not drive changes in Re concentration in the ocean. While the authors do allow for changes in Re burial in anoxic sediments in their model (e.g. Figures 2 and 3), total Re burial is not independent of river Re input but instead is matched to this to maintain ocean steady state. There may be a slight error in Equation 1 (line 164); should the first term after the equals sign be Friv X d187Re-river?

First to clarify, we designed the steady state isotope mass balance model to explore the response of the Re isotope mass balance for the first time. It does not attempt to explore the inter-dependencies of processes in the global Re cycle.

However, the model does allow Re mass in the oceans to vary. For scenarios where global river Re flux increases, Re concentration increases in seawater in the model over the excursion. When Re burial is modified, Re concentrations in seawater can decrease. These changes in [Re] in seawater were not plotted on the figures because concentration of Re in seawater is challenging to reconstruct due to local basin controls, sedimentation rate, and diagenetic factors which will alter [Re]. Hence, our focus on the isotope system.

Making the model more complex and including interdependencies between variables is very appealing, and the reviewer outlines a couple of options here. We note we had already included a Re concentration-dependant output flux, which we have now fully explained (Equations 2-7). However, adding further complexities are not in the scope of our current study. Gathering this Re isotope data was a 2-year endeavour by the first author, set in the backdrop of a prior 4 years of detailed investigation of Re isotopes in modern weathering systems (Dellinger et al., 2023, Dickson et al., 2021, 2024, Wang et al., 2025).

We note that complex models and their prediction of carbon cycle processes are only just including OC_{petro} oxidation (see Hulse and Ridgwell, 2025, *Science*). Even then, these papers have not prescribed any climate drivers and responses to this process, and instead have used it as a forcing. Future work on OC_{petro} oxidation will need to build coupled models that can predict δ^{187}Re isotope records, alongside more established proxies.

Setting aside concerns about magnitude of Re isotopic excursion and Re steady state, the crux of the authors' argument is that a negative Re isotopic excursion on this OAE timescale likely reflects a negative shift in the 187Re value of river inputs. The authors reasonably assign this shift to an increase in weathering of OM-rich rocks. One can assume that within the Monte Carlo simulations there are some that combine the increased river flux of Figure 3A with the negative shift in river Re isotope values of Figure 3B as these two forcings should act in tandem. As such, I wonder if the authors could estimate the mass of CO2 release from the modeled change in river Re flux (and assumptions about Re/C ratios) in addition to the CO2 estimate from temperature sensitivity of oxidative weathering fluxes. The assertion that this all represents a feedback is intriguing, and hopefully will be supported by further study that included mechanistic model of Re mass and isotopic systematics during OM weathering.

This is a really interesting suggestion, and we were inspired to look into this in response to this reviewer and reviewer #3's comments on making more of our modelling approach.

We have looked into this in detail and have concluded that the best approach at this point in time is to **include a new model which captures how weathering intensity influences Re isotope composition of rivers.** In summary, we now use a model, proposed recently by Dickson et al., (2024), to quantify the changes in weathering intensity required to produce shifts in $\delta^{187}Re_{riv}$ that deliver those required by the existing ocean mass balance model (Figure 3A-C). The new results are shown in Figure 3D and this new model explained in detail in the methods.

Dickson et al., (2024) have proposed that weathering intensity could control river δ^{187}Re values in the form of a simple Rayleigh distillation using a set of black shale weathering profiles from the USA and published data from the Mackenzie River basin and another

weathering profile. We take this model (Equations 4 & 5) as a starting point and use a set of parameters based on previous work. We deviate from a starting oxidative weathering intensity of 0.61, which corresponds to the modern day. We find that to recover the magnitude of the T-OAE excursion of -0.22‰, we require weathering intensity to increase to 0.96, equivalent to an increase in oxidative weathering flux of 1.6 times.

This new model addition strengthens our paper because it agrees with an independent approach we already included. In the previous version, we quantified the changes in weathering intensity and flux based on the temperature changes over the T-OAE. This uses the field-based measurements of the apparent temperature sensitivity of OC_{petro} oxidation for a 6-7°C temperature rise. The changes in weathering intensity suggested are very similar to the new weathering model 0^{187}Re assessment.

In addition to these changes, we have more carefully explained the concepts of weathering intensity and the potential for temperature changes to impact global OC_{petro} oxidation rate throughout the manuscript.

There appears to be a typo in line 229.

Spotted – “whether” => weathering.

It would be useful if in Figure 3 the authors not only highlighted the average of the Monte Carlo simulations but also described those simulations that best align with a -0.25‰ isotope excursion.

We like this suggestion – see reply above. This part D of the figure has thus been replaced by the new model.

It is not clear what Figure 2 panels B, C and D bring to the paper beyond what is described in the text.

We have kept these figures, as they show the overall expected behaviour of the OC_{petro} oxidation weathering patterns and rhenium isotope values and are used in the discussion and new model in the modified version. Given there are only a handful of papers on 0^{187}Re at the moment, we think this is useful to include.

Reviewer #3

Introduction:

This is a well-written and well-argued manuscript, with state-of-the-art isotope data (though see my comments on the Os correction) and good documentation of analytical results, including those of geochemical reference materials. The topic is of broad interest and deals with an important issue in geochemistry and Earth’s past climate. It is thus potentially of interest to the broad Nature Communications community.

Thank you for these positive comments. We have revisited the Os isotope correction as we reply in detail below. We thank the referee for their insightful thoughts on this novel isotope system and rock organic carbon weathering.

Arguments:

The key argument is that isotope fractionation of rhenium during weathering of lithologies rich in organic carbon initially release isotopically heavy Re to rivers and the ocean. The more complete weathering is, the more the isotopic composition mimics that of the bulk

lithology. This bulk composition is likely isotopically lighter than ambient seawater. Events in earth history that approach complete Re (and related organic carbon) release, such as brief hyperthermals, should therefore cause a negative excursion in the Re isotope record of seawater. This is what the authors claim to have discovered during the Toarcian OAE (TOAE).

Thank you for this summary, which captures the main variations in the dataset that we have measured, alongside the interpretations we make to explain them.

Model:

The authors then build a quantitative model to explore various transient scenarios. These, for my taste, could be improved. For instance, I urge the authors to develop a coupled Re-Os-C isotope model to quantitatively test which of their scenarios match the observations best. For instance, Dubin and Peucker-Ehrenbrink (2015) have estimated the average Os isotope composition of the types of shales the authors investigated for Re. Together with other literature data, average Re-Os-C concentrations relationships can be established. These isotope tracers can then be coupled to data on the release from weathering sections of black/gray shales. If a model can be devised that matches the observed Re, Os and C isotope data for the TOAE, the study merits publication in Nature Communications. For instance, does the observed increase in 187Os/188Os during the TOAE match the predicted decrease in Re isotope composition and the release of the "missing" C from OCpetro, if an 187Os/188Os of ~2 is used for extra release from organic-rich shales during this event? If not, what isotope composition would result in a match. What does the predicted 013C tell us about the additional OC source? Analogous questions can be asked for carbon and its isotopes. Such a refined/expanded model will have to deal with the change in residence time that is indicated by the low Re concentrations in sediments deposited during the TOAE (lines 121-132), and related effects on the response of the system to perturbation (a la Lasaga & Berner, 1998).

We agree that a coupled Re-Os-C model would be very exciting, and could lead to some novel insight on these processes. However, we disagree that a coupled Re-Os-C is currently within reach and feasible to include as part of our study, and we disagree that its absence detracts from our study and its potential publication at *Nature Communications*. Indeed, the Re isotope system is simpler than the Os and C isotope mass balance and much more attuned to OC_{petro} oxidation (Figure 2).

However, we thank the reviewer for challenging us on this, and have reflected carefully on it. As in our reply to Reviewer #2 above, we now expand our modelling of Re and C. **We now calculate changes in OC_{petro} weathering intensity over the T-OAE via two different model approaches and find they agree**: i) calculating the required changes in weathering intensity to shift river 0^{187}Re values enough to produce a -0.22‰ excursion (Figure 3D; ii) using the apparent temperature sensitivity of OC_{petro} oxidation in kinetically-limited settings to quantify increases in flux and weathering intensity.

The first approach is a major revision to the manuscript. The second was already included, but we now provide the underlying equations in the methods (Equations 6-9). The second approach also confirms the "missing carbon" from Fendley et al., (2024) that caused the T-OAE warming can be supplied by OC_{petro} oxidation.

We have also included new discussion of the Os and C isotope mass balance. This notes that the direction of Os isotope shifts is consistent with enhanced shale weathering (as the reviewer describes above). It also notes that the stable C isotope record is very difficult to interpret.

A new Re-Os-C model, as proposed by the reviewer, would involve a large number of parameters which would need to be explored and tested. While the Re and Os cycles and isotope mass balance have some similarities, and Os isotopes should be sensitive to OC_{petro} weathering, they have notable differences in their sources and sinks which mean that running a model with both isotopes requires constraint on a number of terrestrial and oceanic geophysical and biogeochemical processes. The C isotope mass balance is sensitive to OC_{petro} oxidation, but also requires a whole host of equally important processes (volcanic inputs, C isotope composition of volcanic inputs, carbonate weathering, C isotope composition of carbonates, composition of OC_{petro}, organic matter burial, pCO_2 control on isotope fractionation). Many of these processes are controlled by climatic, tectonic and biological drivers. As such, this is not a simple undertaking.

Indeed, a recently published paper (25 September 2025) makes it clear that these developments would be study in its own right (Hülse and Ridgwell, 2025, *Science*). To our best knowledge, this paper is the first geological carbon cycle model that has OC_{petro} weathering as defined process. This addition and how it can cause instability in the longterm carbon cycle and climate is part of the novelty of this new work, and presumably the reason for its publication in *Science*. However, this modelling work does not include any data, nor examples from the geological record in which to test this new model. Intermediate complexity models (which are the next step beyond a steady state mass balance model we present here) are a significant amount of work.

Our Re isotope record of the T-OAE is one of the major outputs across a 5-year NERC research grant (2018 – 2023) involving the team. Amongst the novelties flagged by this review and others, it can also be a call to arms for the modelling community to better represent these processes in models. We have provided a new proxy from which these complex models can ground their outputs.

It would help the readers to have the data for the TOAE section plotted on the model results (Fig. 3) in order to be able to see how well the observations match the model.

We have decided not to do this, since the time dependency is not what we are trying to model, but instead the magnitude of the excursion. We believe this would constitute an over interpretation of the data. However, please note we have updated the duration of the event to 0.3 Ma in the model based on feedback from Reviewer #1.

I have to admit that I am skeptical of the “weathering intensity” argument. Weathering of gray/black shale lithologies and related release of Re and C is quick and fairly complete, aided by acidic conditions created by the quick oxidation of sedimentary pyrite. I therefore have difficulties accepting the notion of incomplete weathering of such lithologies under almost any near-surface conditions.

We have a huge amount of respect for the reviewer and their contributions to the field, but we strongly disagree with these statements and hope we can convince the editor and reviewers about this.

Rock organic carbon is commonly incompletely oxidised in the near surface weathering zone. This is widespread in erosive environments where mass wasting processes remove material faster than it can be completely weathered. This has been shown in at least two independent scales – first at the rock outcrop scale, and second at the river catchment scale. OC_{petro} can be commonly found in rock outcrops at the surface, and even beneath modern soils, and there are many studies showing this in the European Alps (Soulet et al., 2021); western USA (Blair et al., 2003; Tune et al., 2022); Taiwan (Hemingway et al., 2018); New Zealand (Roylands et al., 2022); and Andes (Dellinger et al., 2023). In contrast, where

denudation and erosion rates are slower, oxidative weathering can proceed for longer periods of time, and near the surface OC_{petro} can be depleted. This was shown in the seminal work by Petsch et al., (2000) in which the reviewer was involved, and may reflect what the reviewer has in mind. But we note that these settings contribute much less to weathering fluxes due to their low denudation rates (Zondervan et al., 2023).

In addition, global river sediments confirm that OC_{petro} oxidation is incomplete, most notably in a seminal paper involving the reviewer – Galy et al,. 2015, *Nature* – which we now cite in the paper (an error to miss out in the original submission). Incompletely weathered OC_{petro} is found in global rivers, and is exported to the oceans in a large flux of 43 $^{+61}/_{-25}$ MtC yr^{-1}. When we compare this to the estimates of OC_{petro} oxidation during weathering, globally, of 68 $^{+16}/_{-6}$ MtC yr^{-1} it suggests that ~60% of the OC_{petro} entering the weathering zone is oxidised (Zondervan et al., 2023).

These two sets of observations, from rock outcrops and river catchments, directly contradict the reviewers assertion above that OC_{petro} is completely weathered at Earth's surface. This means that in the present day, weathering intensity could increase quite notably.

When reflecting on this point, we realised that we did not provide enough text on the modern-day observations of weathering intensity from the river data in the introduction. We have therefore made extensive edits in the second paragraph to introduce the state of knowledge on this important theme, which hopefully clarifies our argument to the reviewer. We have included the Galy et al., 2015, reference and do a better job of explaining the main findings.

For instance, will a 10 degree C change in temperature really matter? I know experiments indicate this (under idealized conditions), and evidence from silicate weathering also indicates temperature dependence. I do think these OM-rich lithologies are special and the matter associated with the organic matter is released quickly and nearly completely.

The published data on the temperature sensitivity of OC_{petro} weathering (Soulet et al., 2021; Roylands et al., 2022) are in-situ field measurements using respiration chambers to measure CO_2 flux in real time, with CO_2 sampling to quantify the rock-derived C contributions using radiocarbon. We are not clear why the reviewer refers to this as "idealised" – they are directly made on the near-surface weathering zone of sedimentary rock outcrops.

The main findings of these two studies agree, one from a Jurassic organic carbon bearing marl in France, and the other from a carbonate-poor Cretaceous mudrock in New Zealand. Both studies return a temperature sensitivity reflecting a Q_{10} ~ 2 (or higher). **In other words, 10 degrees of warming will more than double the flux at those locations.**

On the issue of near complete OC_{petro} weathering – see the reply above – this is simply not a feature of the weathering environment as a whole, and OC_{petro} remains incompletely oxidised across Earth surface environments.

That means that Re that is released during weathering will always mostly reflect the isotopic composition of the source lithology, which will (with fractionation) be linked to the contemporaneous isotope composition of seawater which likely will have varied through time. Such a Re isotope record of paleo-seawater still needs to be reconstructed. At the moment we know little about the Re isotopic composition of continental runoff (and SGD) as only values for the Mackenzie River have been published. To me, it is a global flux issues that is related to the intensity of the hydrologic cycle, rather than a "weathering intensity" issue. I could be convinced otherwise if – for instance – the CIA (or other quantitative measures of "weathering intensity") in the detrital fraction changed during the TOAE. Has it?

Some of the points raised here are of course important (a long-term seawater reconstruction of $\delta^{187}Re$ would be game-changing; a global modern-river assessment of $\delta^{187}Re$ immensely powerful), however, they are out of the scope of our current manuscript. We provide the first paleo-seawater $\delta^{187}Re$ record from a climatic perturbation during the Mesozoic.

Our discussion of weathering intensity derives from our interpretation of the Re isotope shifts and how to best explain them given our current understanding. Inferring the role of temperature change vs hydrological change is interesting in global oxidative weathering fluxes. Unfortunately, we simply don't have data at the moment to pick apart these two drivers. However, we do have data showing a pronounced temperature sensitivity of OC_{petro} oxidation (Soulet et al., 2021; Roylands et al., 2022), and this apparent temperature sensitivity can explain the magnitude of the potential CO_2 release from oxidative weathering during the T-OAE.

There is an additional complication – the role of hydrothermal circulation as an additional player in the global cycle. The authors do not correctly characterize the findings of Miller et al. (2011) regarding submarine HT hydrothermal systems (line 160). These systems are a Re sink, NOT a small source. Miller et al. write that Re is more quickly lost from fluids than Mg, an element that – at zero concentration (i.e. complete removal from seawater) – is used to characterize hydrothermal end-members. Relevant removal fluxes have been calculated by Ghazi et al. (2024, full disclosure – I am a coauthor of that study). To my knowledge submarine LT hydrothermal systems have not yet been investigated for Re isotope composition and concentration.

Apologies that we misrepresented the role of hydrothermal circulation in the manuscript. We should have explained better that the return flux of Re is very small (<1% of the river input), and thus its lever on the Re isotope mass balance muted.

We agree with the reviewer that hydrothermal systems present a net sink of Re, and since hydrothermal fluids can extend to compositions several tenths of per mil higher than seawater, this would indeed imply a net burial of isotopic low Re. In effect, these systems would appear to affect the Re global mass balance in a similar manner to sediment burial. However, we note that the estimated fluxes in Miller et al., 2011, and Ghazi et al., 2024 (now cited in the manuscript – thank you for flagging that useful review paper) show that the removal and return flux are very small.

Additional issues:

Line 35: I don't think it's quite correct to state that "Earth's climate is controlled by variations in the concentration of atmospheric CO_2". Orbital parameters (Milankovic) and global albedo matter as well, among others.

That is true – we have edited the sentence here.

Lines 75/76: "caused by the preferential oxidative weathering of phases enriched in the heavy 187Re isotope" is an interesting characterization as it implies that there are phases in these shales that are enriched in 187Re, and that those phases release their Re first. I don't think of the system in this way because I think isotope fractionation, differential reaction energies and the stability of molecules transporting either 185Re or 187Re are the key. A study of the Re isotope composition extractable organic phases in such shales could provide an answer.

Here in the text we are summarising what is known about the surface cycle of Re and its isotopes during weathering. This statement captures the conclusions drawn by the papers

cited here, namely Miller et al., 2015 and Dellinger et al., 2021. We agree perhaps the terminology could be altered, but we are also aware that Nature Communications is for a wider audience and this is part of the introductory text.

In terms of the extractable organic phases. We welcome more work on sediment extractions because as Dellinger et al., 2021, found, only the “reduced” phase could be extracted using wet chemical techniques. This includes organic matter, but also sulfides, and that paper was careful to discuss this in detail. Here, we cannot extract enough mass from sequential extractions for Re isotopes, and so have focused on changes in the bulk Re isotope composition of these sediments.

Lines 104/5: I think Ravizza et al. (2000) provided the best empirical evidence for weathering feedback operating in the geologic past (in their case the PETM). I also think the link between the Re-Os system and the marine (organic) carbon record, explored by Ravizza & Esser (1993), was very farsighted.

We agree that these papers are interesting and important contributions. However, this paragraph is discussing the T-OAE specifically, and the “missing CO_2” that a recent paper has identified. As such, these papers on the PETM and longer-term Os isotope record are not appropriate to cite here. However, they are very useful in other parts of the discussion on this theme and we have now cited them in the manuscript elsewhere – thank you.

References: many subscripts are missing.

Fixed.

Methods:

Line 470/4: I wonder why the authors did not ash the samples prior to dissolving them in mineral acids. This should have made the whole digestion process much less onerous.

Thank you for this feedback. We were keen to avoid any potential volatilisation of Re during any pre-treatment, which is known to happen during fossil fuel combustion for example, so played it safe. The acid digestion process works well with the reaction vessels we mention.

Line 523 and some data tables: it would be better to replace “ppt” and “ppb” with “pg/g” and “ng/g” to avoid confusion with volumetric measures (parts per... what parts? Weight or volume?)

Thanks. Here we refer to ICP-MS standards, but we agree that we are referring to parts per weight so have changed this.

Line 542: I do not understand why the authors use a fixed 189Os/187Os ratio for the interference correction, and if they use one I don’t understand why they choose a nearly chondritic ratio. These are presumable radiogenic gray shales which would require a lower 189Os/187Os ratio that 8.21944 (the 188Os/187Os value of 5.7504 used at RHUL is a little better, but still much too close to chondritic – a value of ~1.6 would be better). Ideally, the authors used the Os isotope values measured for these samples. I assume these details do not matter in practice because the interferences are likely small, but it would look cleaner if these details were taken into account. How large are the 188Os and 189Os ion beams and the related correction of the 187Re signal?

This is a really insightful comment. The Re isotope system is still relatively “young” and methods are established, but can always be improved.

We have revisited this in our own dataset and explored shifting the $^{189}Os/^{187}Os$ ratio in the interference correction. The average difference between $\delta^{187}Re$ values using a Chrondritic versus non-Chrondritic $^{189}Os/^{187}Os$ value across 10 measurements was 0.0023‰. This is much less than the reported analytical uncertainty of ~0.05‰. We note that the shift can be both positive and negative, indicating no systematic bias in the measurement. As a result of this analysis, we agree further work should establish best practice on this correction, but have kept the methods the same as the published approaches of Miller et al., 2015, Dickson et al., 2021 and Dellinger et al., 2023.

Recommendation: If the authors revise the manuscript and make the model more quantitative it may be suitable for Nature Communications. I'd be happy to review such a revised version.

I don't like anonymous reviews. Please do not hide my identity from the authors.

Bernhard Peucker-Ehrenbrink (WHOI)

We have made significant modifications to the modelling framework and provide more quantitative insight as a result. Thank you again for the thoughtful and detailed review which has helped us improve our work.
